# Dendritic Cell Therapy in Immuno-Oncology: A Potentially Key Component of Anti-Cancer Immunotherapies

**DOI:** 10.3390/cancers18010123

**Published:** 2025-12-30

**Authors:** Emilia Marta Marchelek, Afrodite Nemeth, Sidhesh Mohak, Kamilla Varga, Szilvia Lukacsi, Zsolt Fabian

**Affiliations:** 1 School of Medicine and Dentistry, Faculty of Clinical and Biomedical Sciences, University of Central Lancashire, Fylde Road, Preston PR1 2HE, UK; emmarchelek@lancashire.ac.uk; 2 Translocon Biotechnologies PLC, Akadémia u. 6, 1054 Budapest, Hungary; afrodite.nemeth@translocon.hu (A.N.); kamilla.vogel_varga.dr@translocon.hu (K.V.); szilvia.lukacsi@translocon.hu (S.L.); 3 Department of Medicine, South Texas Health System, McAllen, TX 78503, USA; smohak@mail.sjsm.org

**Keywords:** dendritic cells, antigen presentation, cellular adoptive immunotherapy, cancer immunotherapy, clinical trials

## Abstract

Dendritic cells (DCs) are a heterogeneous population known for antigen presentation and immune modulation, playing a key role in priming a T cell response against pathogens and tumor cells. Despite their putative therapeutic value, their scarcity in peripheral blood limited their direct use in therapeutic applications until recently. The discovery that DCs can be generated from circulating monocytes ex vivo, however, gave a boost of extensive research in the use of DCs in clinical applications. Still, despite the numerous clinical trials, the introduction of DCs in the everyday clinical oncology practice is delayed. In this narrative review, we provide an updated summary of the field covering the theoretical and practical aspects of the concept of the use of DCs in adoptive cellular immunotherapy and the completed or ongoing clinical trials for the use of these species in clinical oncology practice. To better understand the current developments of the field, we included those clinical trial reports that published evaluable data to date. Based on our literature survey, DC-based adoptive cellular therapy is a safe therapeutic intervention with valuable clinical potential. Its widespread implementation, however, is likely delayed due to a number of factors that make meaningful evaluation of clinical trial results complicated. These include the great variety of preclinical trial concepts, difficult and heterogenous patient cohorts, and the diversity of intervention techniques applied. Since these factors might hinder the routine implementation of DC-based applications in the more widespread forms of immunotherapy, one of the urgent short-term future directions seems to be the standardization of the DC-based methodologies.

## Simple Summary

The limited efficacy and severe side effects of traditional anti-tumor therapies prompted a paradigm shift in practical oncology in the past decade. This includes innovative approaches like cancer immunotherapy that aims to exploit the anti-cancer capacity of the innate immune system. Accordingly, numerous new concepts of immune oncology have been developed in recent years, including the use of immune checkpoint inhibitors or genetically engineered T lymphocytes. However, the observed variable clinical effectiveness calls for further developments like those that rely on dendritic cells, master regulators of lymphocyte activation through antigen presentation. Here, we review these efforts, including the ongoing clinical trials and potential future directions of the use of them in clinical practice.

## 1. Introduction

In 2022, cancer was considered to be responsible for one in every six deaths (16.8%) world-wide, representing one of the top three leading causes of premature death in the majority of surveyed countries [1,2]. This accounted for an estimated 20 million new cases and 9.7 million deaths in 2022 [1]. Assuming unchanged cancer rates, a 77% increase in cancer incidence is predicted by 2050, accounting for up to 35 million new cases. This would almost double cancers deaths with the currently available therapeutic tools calling for new approaches.

Indeed, the cellular plasticity and heterogenicity of tumors, in particular the presence of therapy-induced senescence under current therapeutic regimes, makes the efficacy of standard, canonical, not-individualized treatments limited [3,4]. Moreover, canonical therapeutic applications directly deteriorate the endogenous anti-tumor immunity, eliminating a critical factor of natural measures counteracting neoplasms [5]. With our expanding knowledge on genomics, this notion led to a recent paradigm shift from canonical histology-based cancer type-specific modalities to biomarker-centric approaches [6]. The emerging tumor-agnostic philosophy of biomarker-driven approaches in precision medicine attempts to treat patients on the basis of the particular genetic or molecular signatures of their tumor cells, regardless of their histological origin or the anatomical location of the neoplasm [7,8]. Although it also faces challenges due to co-occurring mutations, distinct microenvironments and therapy-induced resistance, this genome-informed and -oriented approach apparently redefines practical oncology, including the rapid implementation of immuno-oncology methodologies [9].

Immuno-oncology is a type of cancer treatment that utilizes, enhances, or modifies a patient’s own immune system to attack cancer cells. In contrast to traditional anti-cancer interventions, that rather deplete immunocompetent species, immuno-oncology treatments focus on a patient’s immune ecosystem to exploit the endogenous immune cells’ capacity to discover and eliminate transformed cells [5,10]. Stimulation of the anti-cancer immune machinery is believed to ensure specificity and immune memory that allows defense against cancer neoantigens even after therapy is discontinued, a critical therapeutic aspect considering the immune system-escaping strategies of cancer cells [4,11].

The first immuno-oncology approaches widely implemented in the clinical practice were centered around immune checkpoint inhibitors that deliberate immune cells paralyzed by cancer species, allowing them to become reactive against neoantigen-harboring cancer cells [11]. While combining immune checkpoint inhibitors with chemotherapy has expanded treatment options for cancers, this approach has shown no clinical benefit in immune desert, also referred to “cold”, neoplasms characterized by the lack of reactive lymphocytes, or in patients with dysfunctional or exhausted T cells [11,12]. To address this challenge, professional antigen-presenting cellular elements of the immune system—like the dendritic cells (DC), capable of bridging the innate and adaptive immune systems—have been explored as a promising platform for novel therapeutic interventions.

In this narrative review, we provide an updated summary of the field covering the theoretical and practical aspects of the concept of the use of DCs in adoptive cellular immunotherapy and the completed or ongoing clinical trials that have published evaluable results.

## 2. Dendritic Cells

### 2.1. Microscopic Characteristics of Dendritic Cells

**Figure 1:**
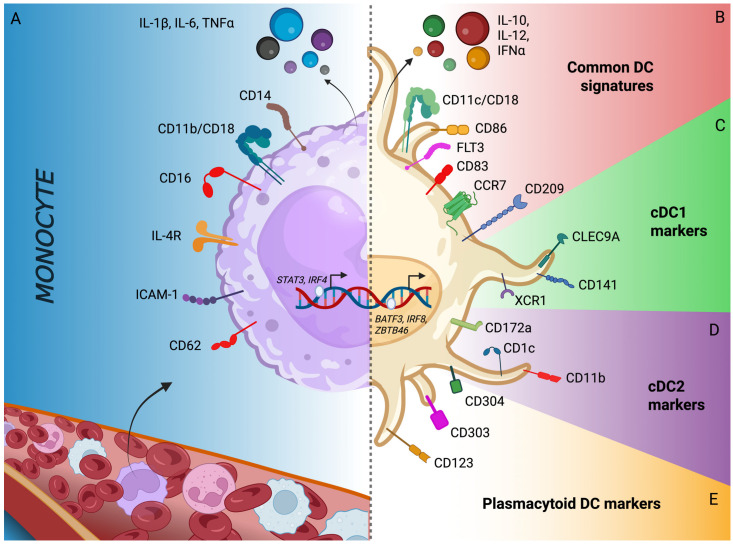

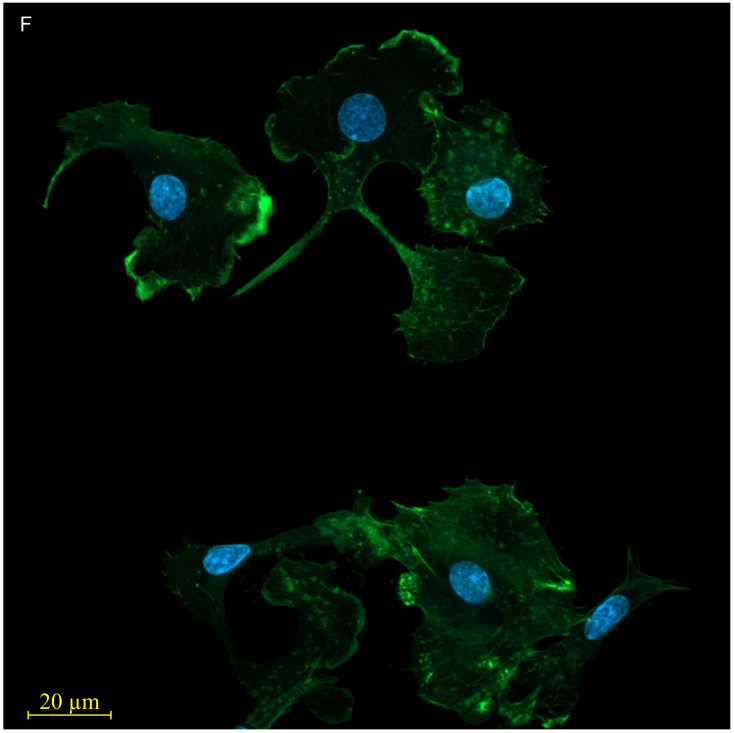
(**A**) Schematic comparison of the molecular and structural characteristics of dendritic cells and monocytes. Circulating monocytes display high expression of either CD14 or CD16, as well as CD11b, ICAM-1, and CD62L, and are characterized by STAT3 and IRF4-driven gene expression. They primarily secrete pro-inflammatory cytokines like IL-1β, IL-6, and TNF-α. (**B**–**E**) In contrast, dendritic cells exhibit CD11c/CD18, CD86, FLT3, CD83, CCR7, and CD209 and are transcriptionally marked by BATF3 (Basic Leucine Zipper ATF-like Transcription Factor 3), IRF8 (Interferon Regulatory Factor 8), and ZBTB46 (Zinc Finger and BTB Domain Containing 46). DCs preferentially secrete IL-10, IL-12, and type I interferons (e.g., IFN-α) along with demonstrating enhanced antigen capture and T cell priming functions. Subset-specific regions further delineate DC phenotypes: (**C**) classical type 1 DCs (cDC1) express CLEC9A, CD141, and XCR1; (**D**) classical type 2 DCs (cDC2) express CD1c, CD172a (SIRPα), and CD11b; (**E**) while plasmacytoid DCs (pDCs) express CD123, CD303, and CD304. The figure was created with Biorender.com. (**F**) Representative image of dendritic cells differentiated from monocytes in vivo. Monocytes were isolated from PBMC using CD14-magnetic beads and differentiated into dendritic cells with the addition of GM-CSF and IL-4. Dendritic cells were collected on the 5th day of differentiation and plated on a fibrinogen-coated coverslip. Microfilaments and nuclei were stained with phalloidin-Alexa488 (Molecular Probes, Invitrogen, Cat A12379, Eugene, OR, USA) and Hoechst 33342 (Thermo Fisher Scientific Cat. #62249, Carlsbad, CA, USA), respectively. Confocal microscopy images were collected using an alpha Plan-Apochromat 63x/1.46 Oil Corr M27 objective of a Zeiss LSM800 Axio Observer Z1/7 system (Carl Zeiss AG, Oberkochen, Germany). Courtesy of Szilvia Lukacsi.

Dendritic cells were first described by Steinman and Cohn as a small population of cells morphologically distinct from known lymphoid species and named after their unique stellate shape (Figure 1) [13]. They were found to be most abundant in mice spleen, accommodating for around 1–1.5% of all nucleated cells, though they were also found in Peyer’s patches and lymph nodes [13].

This new class of cells were shown to have large nuclei and mitochondria with a perinuclear region usually filled with the Golgi apparatus, few small lysosomes with varying contents, and numerous rough and smooth-surfaced vesicles surrounded by a smooth cell surface without any microprojections. The most distinguishing characteristic of dendritic cells was reported to be their relatively electron-lucent ground cytoplasm, which sets them apart from other nucleated leukocytes, especially from lymphocytes (Figure 1) [13].

### 2.2. Ontogeny of Dendritic Cells

**Figure 2:**
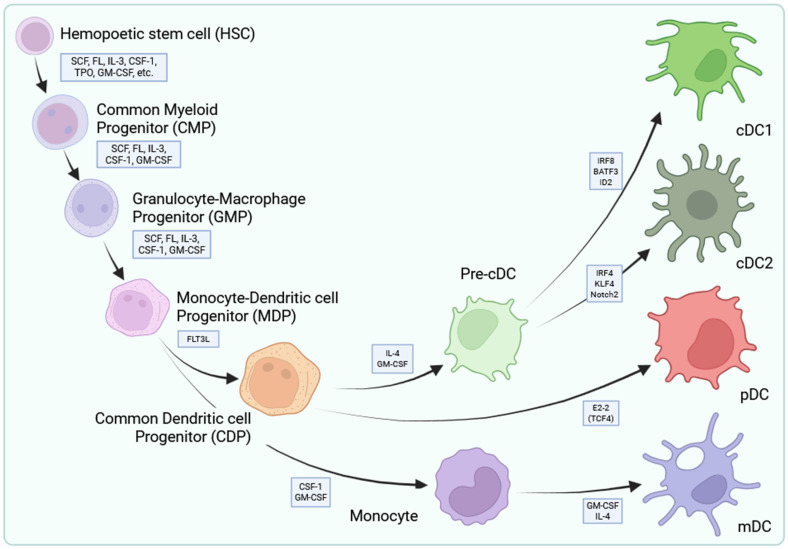
Histological origin of dendritic cells. Hematopoietic stem cells (HSCs) in the bone marrow differentiate into granulocyte-macrophage progenitors (GMPs) via intermediate species termed common myeloid progenitors (CMPs). Under the influence of specific cytokines (SCF, FL, IL-3, CSF-1, GM-CSF), GMPs give rise to monocyte-dendritic cell progenitors (MDPs) from which differentiation can branch into two main lineages: the monocyte lineage, which gives rise to monocyte-derived dendritic cells (mDCs) and macrophages in peripheral tissues via monocytes, and the conventional DC lineage, in which MDPs differentiate into common dendritic cell precursors (CDP). These can directly give rise to plasmacytoid dendritic cells (pDCs) through E2-2 (TCF4)-dependent transcriptional programming or to pre-conventional dendritic cells (pre-cDCs) upon the activity of FLT3L signaling which subsequently differentiate into cDC1 or cDC2 subsets. The figure was created with Biorender.com.

DC development originates in the bone marrow from hematopoietic stem cells [14]. Granulocyte-monocyte-DC progenitors give rise to monocyte/DC progenitors, which have the potential to develop into all DC subsets [14] (Figure 2). Analyses of their molecular makeup revealed that typical DCs show elevated expression of major histocompatibility complex class II molecules and integrin CD11c but do not display the typical surface markers found on B cells, T cells, macrophages, or granulocytes (Figure 1) [15,16]. Further studies revealed that DCs can be divided into functionally different subsets like the CD11c^+^ CD123^−^ conventional myeloid and CD11c^−^ CD123^+^ plasmacytoid lymphoid DCs [17,18]. When monocyte/DC progenitors differentiate into common DC progenitors, first, they lose their capacity to become monocytes [14]. This species, then, can differentiate into either plasmacytoid DCs or circulating pre-conventional DC progenitors. The latter ones can further differentiate into conventional DCs (cDC) by lineage-determining transcription factor-orchestrated processes [14]. cDC can be further classified into two subsets, conventional type 1 DCs (cDC1) expressing CD141 (BCDA3) and conventional type 2 DCs (cDC2) expressing CD1c (BDCA1) and CD11b (Figure 2) [19,20,21]. The development of the former one is strictly dependent on the basic leucine zipper ATF-like transcription factor 3 (BATF3), while the development of the latter one is linked to transcription factors Interferon Regulatory Factor 2 and -4 (IRF2 and IRF4) [22,23,24].

### 2.3. Antigen Capture and Processing by Dendritic Cells

**Figure 3:**
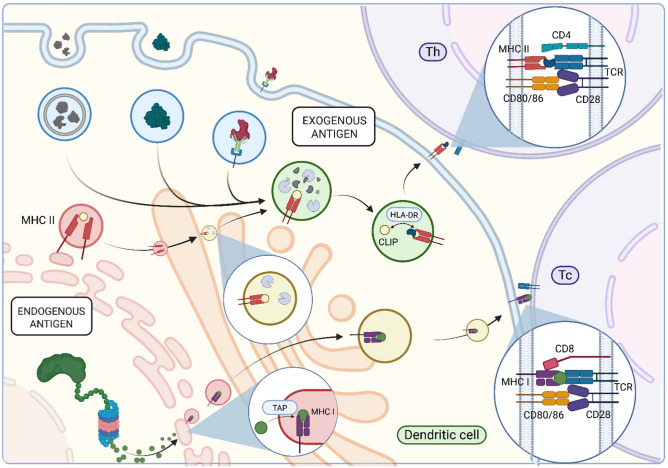
Antigen capture, processing, and presentation by dendritic cells. Dendritic cells have the capacity to present both endo- and exogenous antigens, thereby enabling immune surveillance against extracellular and intracellular factors. During the exogenous antigen presentation, major histocompatibility complex class II (MHC II) molecules are assembled in the endoplasmic reticulum (ER) in association with the invariant chain containing the class II-associated invariant chain peptide (CLIP) and form late endosomes. Dendritic cells can internalize exogenous antigens through selective phagocytosis, macropinocytosis, or receptor-mediated endocytosis. Antigen-transporting primary endosomes fuse with MHC II-marked late endosomes where the proteolytic degradation of the engulfed cargo occurs. Exchange of CLIP for peptides generated from processed antigens is mediated by HLA-DM, resulting in stable MHC II-peptide complexes that are, eventually, delivered to the cell membrane for presentation to CD4^+^ T helper (Th) cells. In the endogenous pathway, in contrast, cytosolic proteins are degraded by the proteasome and the resulting peptides are transported into the ER via transporter associated with antigen processing (TAP) and loaded onto MHC I proteins. The complex, then, is transported to the cell surface to be recognized by CD8^+^ cytotoxic T (Tc) cells. In both cases, effective T cell activation requires additional costimulatory signaling like CD80/86-CD28 connection. The figure was created with Biorender.com.

Physiologically, DCs play the central role in triggering immune reactions via the process called antigen presentation (Figure 3). Differentiated DCs capture, process, and present antigens to T cells [25]. In case of exogenous antigens, this process begins with the internalization of antigens via non-selective macropinocytosis, selective phagocytosis (e.g., of apoptotic bodies or opsonized particles), or receptor-mediated endocytosis (targeting smaller or soluble antigens) [13,26]. The constitutive, non-selective macropinocytosis is mediated by aquaporins like AQP3 and AQP7 that are expressed in immature DCs and ensure the uptake of large quantities of solutes that may contain soluble extracellular antigens [27]. In contrast, selective antigen uptake is mediated by a variety of receptors, including Fc receptors, complement receptors, C-type lectins, and scavenger receptors [26,28,29,30].

Following antigen internalization, dendritic cells generate macropinosomes, phagosomes, and endosomes. These early endocytic vesicles subsequently fuse with late endosomal compartments containing MHC II molecules, initiating the degradation of internalized proteins [31,32]. Within these vesicles, the chaperone HLA-DM catalyzes the exchange of the MHC II-associated CLIP fragment for high-affinity antigenic peptides [33]. Once bound, the MHC II-peptide complex is transported to the plasma membrane for antigen presentation [34].

In case of endogenous proteinaceous antigens, polypeptides undergo degradation via the ubiquitin-proteasome pathway, and the resulting peptide fragments are transported to the endoplasmic reticulum (ER) by the transporter associated with antigen processing (TAP) molecule [35,36]. Peptides of appropriate size, usually 8 to 10 amino acids, and sequence bind to MHC I molecules with the assistance of the peptide-loading complex which includes TAP and the chaperon Tapasin proteins [37]. The MHC I-peptide complex is then transferred by vesicular transport, first, through the Golgi and, then, via secretory vesicles to the plasma membrane (Figure 3) [38].

### 2.4. Antigen Presentation by Dendritic Cells

Matured, antigen-presenting DCs facilitate T cell activation through multiple signals in the lymph nodes. The MHC II-peptide complex activates CD4^+^ T cells (helper T cells, T_h_), while the MHC I-peptide complex activates CD8^+^ T cells (cytotoxic T cells, T_c_) through their T cell receptors (TCR) [39,40]. These interactions are further stabilized by CD4 and CD8 co-receptors and additional stimulatory factors like CD80-CD28 engagement [41]. In terms of antigen presentation, dendritic cells have a unique ability to present exogenous antigens on MHC I, a process termed cross-presentation. This process allows DCs to activate CD8^+^ T cells against exogenous antigens like neoantigens of cancer cells by protecting exogenous antigens from immediate degradation. Upon cross-presentation, antigens stored within endosomal compartments are degraded by intra-endosomal proteases or, alternatively, transported to the cytosol and processed by immunoproteasomes [42,43,44,45]. From the cytosolic pathway, these peptides are TAP-dependently imported back into endosomal compartments or the ER for loading onto MHC I molecules, after which the peptide-MHC I complex is transferred to the plasma membrane [46,47]. Besides these physical interactions, DC-derived cytokines are also important regulators of the DC-triggered differentiation of T cells into distinct T_h_ or T_c_ subtypes (recently reviewed in [48]).

During their lifespan, DCs exist in immature and mature states. In the immature stage, they are scattered through peripheral non-lymphoid tissues and act as the body’s first line of defense, constantly monitoring the local environment [49]. They exhibit low expression of MHC I and -II, and the costimulatory molecules CD80, CD83, and CD86 [49]. Being exposed to pathogens, they pick them up and process their antigens to eventually move to lymphoid tissues. Through maturation, cDCs upregulate expression of CD80, CD83, and CD86; increase production of immunostimulatory cytokines (IL-12, TNF-alpha, IL-10); and increase surface stability and expression of antigenic peptide-MHC complexes [49]. Consequently, cDCs lose their ability to pick up further antigens and eventually become mature immunostimulatory cells and strong activators of naïve CD4^+^ and CD8^+^ T cells via direct antigen presentation [49]. As one can expect, the gene expression patterns dynamically change during the complex process of dendritic cell maturation [50]. CD11c^−^ DCs mature using CD40L and IL-3 and do not upregulate genes like *TUBA1A*, *TUBB*, *SPP1*, or *GPNMB* that are typically associated with mature conventional DCs [50]. In accordance, most DC-associated genes such as *MMP12*, *Z39Ig*, *GPNMB*, and *SPP1* are low in immature DCs and increase with maturation. Only a few genes, like *CCL17* in monocyte-derived DCs (detailed below), *HLA-DRA* in all DC subclasses, or *CD1B* in monocytes and CD1a^+^ DCs, have been reported to be constitutively overexpressed in immature DC species [50].

Following exposure to activators like microbes, cDCs secrete pro-inflammatory cytokines like IL-12, IL-23, and IL-10 that, along with surface molecules like OX40-L or ICOS-l, trigger maturation of naïve T cells into their effector Th1, Th2, Treg, or Th17 counterparts [51,52,53,54,55]. IL-12 plays a pivotal role in cDC physiology as it allows them to mature into antigen-presenting cells and elicit their functions [56]. Expression of some of these cytokines, however, shows cell-type specificity like the IL-10 that has been reported to be expressed only in interstitial DCs [57].

cDC1s specialize in cross-presentation and exogenous antigen presentation on MHC class I molecules to activate naïve CD8^+^ T cells that can develop into cytotoxic T lymphocytes. For this activity, cDC1s uniquely express endocytic receptor CLEC9A and chemokine receptor XCR1, which enables antigen capturing, cross-presentation, and T cell activation, respectively [22,58]. cDC2s, in contrast, primarily present antigens on MHC class II molecules to prime naïve CD4^+^ T cells and provide the necessary costimulatory signals [19]. Data indicate that IRF4 regulates the antigen processing capacity and the MHC class II-linked antigen presentation of cDC2s, leading to more robust CD4^+^ T cell proliferation compared to cDC1s [59]. cDC2 also promotes Th2 and Th17 response in contrast to cDC1s that, under standard physiological conditions, do not promote Th2 responses [60].

Plasmacytoid dendritic cells (pDC) do not express CD11c in humans but are positive for CD123 and CD303 [61,62,63]. The key transcription factor responsible for pDC development is the E-protein transcription factor E2-2 [64]. pDCs express a range of Toll-like receptors (TLRs), including TLR7/8 and TLR9, which enable them to detect viral nucleic acids, including single-stranded RNAs and unmethylated CpG-rich DNAs [64]. One of the pDC key functions is the rapid secretion of type I interferons (IFN) and promotion of survival of antigen-activated T cells [64,65]. It is believed that the rapid kinetics of pDC activation is mediated by their high secretory capacity and elevated basal expression of transcription factor IRF7 [66]. In contrast to cDCs, pDCs have minimal antigen-presenting abilities [56,67].

The significantly different gene expression profiles of conventional and plasmacytoid DCs underpin the functional differences [50]. Genes highly expressed in myeloid cDCs like *SPP1* (for osteopontin protein), *FTL* (for ferritin L-chain), *TUBA1A* and *TUBB* (for α- and β-tubulin), *ENO1* (for enolase-1), *ANXA2* (for annexin A2), *CCL2*, *CCL13* (for MCP-1 and MCP-4), and *LAMP3* (for DC-LAMP protein) are associated with antigen uptake and processing, lysosomal function, chemotaxis, and cytoskeletal remodeling, reflecting the phagocytic and migratory capacity of these species [50]. Genes preferentially expressed in pDCs, like *IRF4*, in contrast, are rather essential for Type 1 interferon production.

Thus, our current understanding is that DCs play a pivotal role in triggering various immune cascades, while they can also contribute to the maintenance of the inflammatory state via promotion of cytokine-mediated pro-inflammatory signals. These characteristics make them particularly attractive for cellular immunomodulatory therapies.

## 3. DC Isolation and Culturing

**Figure 4:**
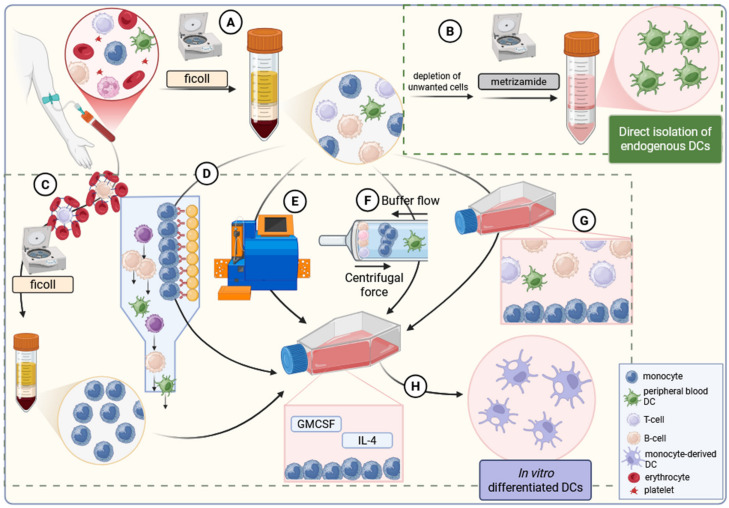
Strategies for dendritic cell enrichment from peripheral blood. (**A**) Peripheral blood mononuclear cells (PBMC) can be isolated from whole blood via ficoll density gradient centrifugation. (**B**) From PBMC, circulating DCs can be enriched following the removal of mono- and lymphocytes and metrizamide-based density centrifugation. Alternatively, DCs can be generated in vitro from monocytes. Monocytes can be purified from PBMC via (**C**) erythrocyte rosetting when lymphocytes are crosslinked to erythrocytes for subsequent removal, (**D**) by immunomagnetic separation using either negative or positive selection strategies, or (**E**) with fluorescent activated cell sorting. Monocyte enrichment can also be achieved through (**F**) centrifugal elutrition or (**G**) adherence-based selection of monocytes after the removal of non-adherent PBMCs. (**H**) Isolated monocytes, then, can be differentiated into dendritic cells resulting in a high yield of DCs. The figure was created with Biorender.com.

Since DCs represent a small proportion of human blood mononuclear cells; their therapeutic use is believed to require enrichment [68]. Historically, first, this was achieved from peripheral blood mononuclear cells (PBMC) by their separation from lymphocytes, monocytes, and macrophages followed by buoyant density gradient centrifugation using metrizamide as density medium (Figure 4) [68]. DC populations can also be generated from monocyte populations of peripheral blood that can be obtained via various methods. On one hand, monocytes can be purified from PBMC using immunorosetting techniques, which deplete unwanted lymphocyte populations via erythrocyte crosslinking. On the other, they can be purified through cell surface marker-based purification approaches that typically use the combination of antibody-mediated immunomagnetic selections and sterile, closed-system flow cytometric sorting. Alternatively, the size, density, and surface-adherent characteristics of monocytes can also be exploited via counter-flow centrifugal elutriation and their exposure to tissue culture plastics under standard conditions, respectively (Figure 4) [68,69]. Surveying available clinical trials reveals that monocyte enrichment is most commonly performed either by adherence or by counter-flow elutriation, which together constitute the two dominant approaches in current clinical practice. It is noteworthy, however, that data also indicate that preparation techniques influence the immunogenic potency of in vitro generated DCs. Indeed, autologous DC preparations derived from plastic-adherent monocyte populations induced superior T cell proliferation than those DCs that were differentiated from monocytes following CD14^+^ selection [70,71]. Incubating them in the presence of GM-CSF and IL-4 in vitro, isolated monocytes differentiate into immature DCs within 5 to 7 days. Since the final number of DCs depends entirely on the number of monocytes that are far more numerous in the peripheral blood than DCs, this method produces much higher yields of DCs in comparison to isolating naturally occurring DCs [68]. Independently of the chosen technique, CD83 is routinely used to assess enrichment of mature DC populations [72].

Following differentiation, exposure to proinflammatory cytokines, like TNF-alpha and IL-1beta or TLR agonists (e.g., LPS, Poly-I:C, R848), for an additional 1–2 days allows differentiated species to obtain their full antigen-presenting functionality, a process termed maturation [68]. In vitro, DCs can be modified before or during their maturation process to enhance their functional properties. Indeed, exposing murine bone marrow-derived DCs to CCL3 and CCL19 leads to an increased and preserved antigen uptake and processing capacity, even after maturation, which then allows a more robust and durable immune response [73]. Moreover, because DCs can phagocytose apoptotic tumor cells and thus allow tumor antigen cross-presentation, they are believed to be able to exert anti-tumor responses following their preconditioning to tumor antigens [74]. Technically, this can be achieved by peptide-pulsing, co-incubation with tumor cells, or transfection with tumor antigen-related mRNAs [75]. The latter method involves in vitro-transcribed mRNAs encoding tumor-associated antigens that, following their intracellular translation and processing, DCs can present on their surface [76]. This MHC-mediated display of immunogenic tumor-specific peptides is believed to enable DCs to effectively activate tumor-specific T cells, initiating a targeted immune response against neoplasms. Another potential benefit of preconditioning is the ability to enhance DC migration from peripheral tissues to lymphoid tissues. It has been shown that PGE2-preconditioned human monocyte-derived DCs have significantly higher CCR7 expression accompanied by an enhanced chemotaxis towards lymph node chemokines CCL19/CCL21 [74,77]. This has been exploited in a number of clinical trials using ex vivo-matured DC preparations [78,79,80,81,82].

It is noteworthy, however, that cytokine dosages and quality-control criteria are significantly inconsistent across publications. Similarly, the reported levels of differentiation exhibit up to a two-fold variation, and maturating stimuli also substantially differ between studies. In addition, some studies describe marker expression qualitatively (e.g., ‘low,’ ‘moderate,’ or ‘high’), while others provide a wide range of phenotypic or functional attributes. Sterility requirements are similarly inconsistent; trials usually report results of mycoplasma, endotoxin, Gram staining, or general bacterial contamination without providing specifics of the assays used.

After isolating and culturing, DCs are reported to be suitable for being stored frozen without losing their functionality. Although several cryoprotectants were tested, 10% dimethylsulfoxide (DMSO)-containing freezing media remains the most commonly used one, sometimes reduced to 5% to minimize DMSO-induced cytotoxicity [83,84,85,86]. The cryopreservation vehicle generally relies on autologous serum-based formulations, sometimes combined with culture media or human serum albumin to support cellular stability during freezing [87,88,89,90]. To further improve post-thaw viability, additives, like glucose in 2–5% final concentration or 12% working concentration of hydroxymethyl starch, are also used alongside DMSO even in GMP-compatible settings [78,91,92,93]. There are considerable efforts to eliminate DMSO as a cryopreservative; studies have evaluated trehalose and a combination of sucrose, isoleucin, and glycerol, but these approaches remain largely preclinical [94,95]. Despite the relative variety in terms of the cryopreservation medium components, protocols are consistent with the 1 °C per minute freezing until reaching −80 °C. Although the literature data indicate that cryopreserved DCs retain both viability and functionality for at least 24 months even at −80 °C in a solution containing 6% hydroxyethyl starch, 5% dimethyl sulfoxide, and 4% human serum albumin, the industrial standard long-term storage is in liquid nitrogen [96].

Being able to culture them in vitro and successively store them for long terms supported the exploration of the use of DCs as tools of the cell-based immunotherapies [73].

## 4. Therapeutic Use of DCs

Exploration of antigen-presenting cell (APC)-based therapies has led to the registration of the first FDA-approved cellular advanced therapy medicinal product (ATMP) that has the goal to boost the anti-tumor immunity via autologous APCs. Sipuleucel-T is an autologous active cellular immunotherapy approved for the treatment of metastatic castration-resistant prostate cancer [97]. The vaccine consists of the patient’s own peripheral blood mononuclear cells, including APCs, which are ex vivo activated using the recombinant PA2024, a fusion protein of prostatic acid phosphatase (a prostate tumor-associated antigen) and GM-CSF [97]. Since the approval of Sipuleucel-T, a number of clinical trials have been exploring the use of antigen-presenting cell-based solutions either as monotherapy or in combination with more canonical chemo-, radio-, or immune checkpoint inhibitor therapies. A comprehensive list of clinical trials that have already published results is provided in Table S1. More than two hundred clinical trials on the use of APCs have been published or registered to date, and more than 60% have published evaluable data. Despite the diversity in targeted neoplasms and clinical scenarios, there are characteristic principles they commonly share.

One of these is that the vast majority of trials used monocyte-derived species generated by incubation using GM-CSF and IL-4. The mode of monocyte enrichment, concentration of the applied cytokines, and term of differentiation, however, are very diverse, and the latter two factors are, apparently, rather empirical. Monocytes are purified from venous blood-derived mononuclear cells most commonly by exploiting their plastic adherent nature. The second most common technique for monocyte isolation is their immunomagnetic separation exploiting their CD14 positivity, while in a small number of trials monocytes are separated from PBMC by elutriation (Table S1).

In terms of their maturation, protocols are similarly diverse, though they can be classified into few major groups. In a number of trials, DCs are incubated with tumor cells during their maturation, aiming to engage them toward the patient’s own tumor cells. This approach has been tested for various cancers with varied outcomes. In a phase I/II trial with stage IV or recurrent melanoma patients, for instance, administration of autologous DCs co-cultured with autologous tumor cells showed excellent long-term outcomes with median overall survival of almost 50 months, with more than 40% of the patients being alive at five years [98]. This concept led to remarkable clinical outcomes in malignant glioma cases, almost doubling the median overall survival time, but failed to deliver similar results in other trials targeting metastatic conditions, probably reflecting on the dynamic nature of cell surface neoantigen repertoire of cancer cells [99,100,101,102].

One of the possible solutions to address this challenge is the use of autologous tumor cell lysates for priming the therapeutic DCs population. Interestingly, while this technique seems to be working in melanomas and glioblastomas, it did not improve the clinical outcome of metastatic conditions, for instance, in renal cell carcinomas where this model was studied extensively [103,104,105].

Similarly, although it significantly decreased PSA levels in prostate cancer patients, autologous DCs preconditioned with apoptotic tumor cell lysates did not improve overall survival despite that it was reported to induce significant CD4^+^ and CD8^+^ T cell proliferation without affecting immunosuppressive FOXP3^+^ regulatory T cells [106]. To improve immunogenicity, autologous tumor cell lysates coupled to immunogenic carriers like yeast wall particles have also been tested and were found to dramatically improve survival of clinically disease-free, stage III/IV melanoma patients upon their vaccination with autologous DCs preconditioned with yeast wall particle-linked lysate, suggesting a possible direction to improve tumor lysate-based protocols [107].

Another regular approach to boost the effectiveness of both the conventional and APC-mediated therapies is the implementation of DC vaccines in traditional chemo- and/or radiotherapy regimes. This has been studied in a number of trials using autologous tumor lysate-pulsed DCs in combination with radio- or chemotherapy, showing successful induction of tumor-specific immunity in glioblastoma patients, resulting in longer survival without major adverse events [108,109,110,111].

Another major class of methods to direct DCs against the desired neoplasm is their load with predefined tumor-related proteins or peptides upon their maturation process. These include a wide range of putative tumor antigens from naturally occurring endogenous biomarkers through their fusion formats to synthetic tumor-associated peptides. The immunogenic nature of these peptides has been confirmed in a number of trials. Indeed, successful induction of T cell-mediated anti-tumor immunogenicity has been recorded upon the use of DCs loaded with peptides from the immune stimulatory T cell receptor alternate reading frame protein (TARP) in prostate cancer patients [112,113,114]. Similarly, monocyte-derived, conventional, type 1-polarized DCs loaded with synthetic glioma-associated antigen peptides evoked anti-tumor immune responses in nearly two-thirds of malignant glioma patients [115]. This strategy has been widely studied, targeting well-known tumor biomarkers like HER-2, MUC-1, or p53.

HER-2-pulsed DCs have been reported to show clinical benefits in patients bearing HER-2-positive breast cancer, reaching nearly 20% complete elimination of disease or, in the presence of residual cancers, repression of HER-2 expression [116]. Even in metastatic breast neoplasms, administration of HER-2-pulsed DCs showed clinical benefits accompanied with immune activation against HER-2 in around one-third of the patients [117]. An independent randomized double-blind phase II trial involving a larger cohort of glioblastoma patients led to comparable conclusions using autologous DCs pulsed with six synthetic peptide epitopes derived from glioblastoma-associated antigens MAGE-1, IL13Rα2, AIM-2, TRP-2, gp100, and HER-2 [118]. Study results also indicated correlation between the progression-free survival and the tumor HLA pattern, suggesting that HLA profiling of the target tumor tissues might improve the efficiency of DC-based therapeutic approaches.

Interestingly, however, clinical trials applying the preconditioning strategy using HER-2, CEA, WT1, MAGE2, and Survivin antigen-overexpressing apoptotic tumor cells did not show comparable efficiency in lung cancer patients, raising the question of whether implementation of the use of DCs can deliver similar clinical advantages in distinct cancer types [119]. Indeed, when metastatic colorectal cancer patients were exposed to DC isolates expressing MUC-1, an O-glycosylated membrane-bound protein that plays an essential role in forming protective mucous barriers on epithelial surfaces, DC-treated patients had higher survival rates [120]. However, when another MUC-1-preconditioned DC isolate was used in ovarian cancer patients, no significant improvement of their progression-free survival was observed [121]. Similarly, modest or no clear, universal clinical benefits have been recoded upon the use of TP53-overexpressing DC in both a small cell lung cancer and a heterogenous cancer cohort trial, either as a standalone treatment or along with the indoleamine 2,3-dioxygenase (IDO) inhibitor indoximod, respectively [122,123].

A variation of the peptide-mediated maturation of DCs is the forced endogenous expression of the desired tumor-associated protein following electroporation, transfection, or transduction. An example of these studies examined the use of antigen-loaded autologous dendritic cells in combination with traditional cytostatic monotherapy in castration-resistant metastatic prostate cancer [124,125]. In this trial, autologous monocytes were matured into DCs by transfections with mRNAs encoding PSA, the prostate tumor-associated antigen Prostatic Acid Phosphatase (PAP), Survivin, and hTERT. Patients were treated either with chemotherapy alone or in combination with DCs, and data indicate that 23% more patients showed decline in serum prostate-specific antigen (PSA) levels, a liquid biopsy measure of the tumor progression, in response to the combinatorial autologous DC treatment compared to chemotherapy alone [124,125].

Besides classical radio- and/or chemotherapy, more recent platforms have also been tested in combination with DCs. For instance, autologous DCs targeting vascular antigens in combination with the tyrosine kinase inhibitor dasatinib were tested in patients suffering from immune checkpoint inhibitor-refractory melanoma, showing nearly half of the patients showed immunological and clinical responses [126]. In contrast, however, clinical evaluation of the putative synergy between the anti-PD-L1 monoclonal antibody-based immunotherapy and the use of autologous DCs in metastatic colorectal cancer patients resulted in only modest clinical benefits, highlighting the importance of further refinements of clinical criteria and type of DC isolates to be applied upon the use of DCs in oncology treatments [80].

Finally, some study results suggest that the route of administration of DC isolates may also impact the clinical response, apparently, in a cancer-type specific manner. Indeed, intratumoral DC injections of soft tissue sarcomas have been reported to lead to long-term disease control [127]. The same application of pre-activated allogeneic DCs in patients suffering from metastatic renal cell carcinoma, however, led to ambiguous results, with some patients having increased CD8^+^ T cell infiltration in the tumors and others showing no objective tumor response at all [104].

## 5. Discussion

Advances in anti-cancer immunotherapy have fundamentally been reforming clinical oncology in the past decade. At the frontline of this paradigm shift is the implementation of immune checkpoint inhibitors (ICI) into the clinical practice that aim to uplift the tumor cell-mediated molecular blockade on the endogenous anti-tumor activity of the patient’s immune system [128]. Despite the great results of their use in certain clinical settings, like some hematological tumors or melanomas, the introduction of ICI therapy did not solve the problem of human neoplasms like gliomas or pancreatic cancer [129,130,131,132]. Moreover, even some initially responding conditions develop resistance to ICI over time, underlining the limitations of this approach [133]. In addition, while their use in monotherapy shows clinical benefit in just approximately 20% of the patients, the combinatorial use of ICI is often limited due to the higher risk of severe, sometime fatal, autoimmune complications that can occur even after months of their administration [134,135]. These challenges suggest that to achieve the effective but safe activation of the cytotoxic T lymphocyte-mediated anti-tumor activity in distinct clinical scenarios, complementary approaches might be implemented in the ICI-based therapeutic regimes. These could be the APC-based approaches that are predominantly relied on for the use of various dendritic cell preparations.

Indeed, the central role of dendritic cells in the activation of immune effector species prompted the concept of their use in clinical applications. The relative scarcity of them in the circulation, however, hindered the swift implementation of endogenous DCs in clinical immunotherapy, calling for the development of in vitro solutions like the monocyte-derived autologous DC preparations. These have been extensively studied in both adult and pediatric clinical settings in recent years with promising, although sometimes ambiguous, results, so their genuine efficacy is still to be determined [136,137,138,139,140]. One of the most important steps in this task seems to be the standardization of protocols applied since current clinical data are incompatible to one another due to the diversity of the application routes, patient cohorts, and, probably most importantly, the DC preparations used in past clinical trials.

Indeed, the more accurate identification of DC subtypes suitable for anti-cancer applications in distinct clinical settings is a critical question to be solved. Increased amounts of plasmacytoid DCs, for instance, seem to correlate with better prognoses among pancreatic cancer patients, while they are rather associated with worse prognosis in breast cancer, hepatocellular cancer, melanoma, and ovarian cancer [141,142,143,144,145]. However, observations that worse prognoses show association with the expression of inhibitory markers like the lymphocyte-activation gene 3 (LAG-3), PD-1, and CTLA-4, immunosuppressive cytokines like IL-10 and TGF-β, and the accumulation of regulatory T cells raise the question of whether the use of ICI in these cases supports APC-mediated actions and enhances the therapeutic effect of ex vivo-raised APCs [141,146,147,148]. This is a particularly interesting question considering the observations that the long-term DC-mediated anti-tumor immunity, at least in certain clinical settings, is, apparently, rather mediated by NK- than T cell populations upon some combinatorial treatments [149].

Moreover, while the majority of contemporary studies use in vitro-raised, monocyte-derived DCs, some data suggest that neogenesis of DCs for therapeutic purposes is not always the clinically most efficient solution. A randomized trial that aimed to evaluate clinical and immune responses to a multipeptide-preconditioned DC preparation in melanoma patients showed that the use of in vitro-generated DCs along with the administration of GM-CSF produced stronger T cell responses and more clinical tumor regressions compared to the standalone administration of dendritic cells [150]. The potential benefits of the mobilization of endogenous DC populations have further been strengthened by the report showing enhanced T cell and antibody responses upon the use of FMS-like tyrosine kinase 3 ligand (FLT3L), a known cytokine stimulating DC progenitors, in patients with resected melanoma [151,152,153]. These data indicate that more details are needed on the environmental factors that support the genesis of DCs that can, then, effectively support anti-tumor immunity.

Independently of their origin, the ultimate goal of the use DCs is the activation of the anti-neoplastic immunity via antigen presentation to immunocompetent elements. One of the major disadvantages of the mobilization of endogenous DCs is the lack of control over their tumor antigen recognition, internalization, and presentation. To address this challenge, various efforts have been made to expose ex vivo-raised DCs to a wide range of possible antigens, from purified known tumor antigens to complex tumor cell lysates [102,154]. The disadvantage of the former method is that it cannot adapt to the dynamic antigen landscape of tumors, while the latter solution requires tumor material. These issues call for further developments like the one recently published by Ghasemi et al., who successfully enhanced anti-tumor immunity in melanoma mouse models using DC progenitors engineered to internalize cancer cell-released extracellular vesicles for facilitating more effective in vivo tumor antigen presentation without the need to know the exact tumor antigens or have cancerous tissue to be lysed available [155].

## 6. Conclusions

Despite the broadly accepted practical potential of the concept of anti-cancer immunotherapy, its widespread clinical breakthrough is yet to fully unfold. It seems to be, now, clear that the use of measures like immune checkpoint inhibitors or genetically engineered T lymphocytes in monotherapy have limitations that shifted the focus of current innovations toward the combinatorial use of immunotherapy tools. This fueled a number of trials on dendritic cells resulting in variable, sometime even contradicting reports on the clinical outcome. Reviewing published data, apparently this is due to a number of factors, including the diversity of clinical trial concepts, difficult and often very limited patient cohorts, or the incomparable techniques of interventions applied [79,122,156]. Considering the heterogeneous nature of both the endogenous and ex vivo-generated dendritic cell populations, the latter issue seems to be the key problem for the appropriate assessment of the clinical benefit of DCs in immunotherapy. This challenge would certainly profit from an optimized and, at least to a certain extent, standardized consensual protocol of ex vivo manipulations including isolation, differentiation, and maturation. In addition, current discrepancies in quality-control assessments and sterility testing in monocyte-derived dendritic cell-based therapies underscore the critical need for harmonized manufacturing protocols and standardized phenotypic and functional characterization panels that would enhance reproducibility and enable more meaningful cross-trial comparisons.

## Data Availability

No new data were created for this work.

## Supplementary Materials

The following supporting information can be downloaded at https://www.mdpi.com/article/10.3390/cancers18010123/s1. Table S1: Summary of clinical trials investigating the use of dendritic cells in clinical settings reported with accessible study results to date [157,158,159,160,161,162,163,164,165,166,167,168,169,170,171,172,173,174,175,176,177,178,179,180,181,182,183,184,185,186,187,188,189,190,191,192,193,194,195,196,197,198,199,200,201,202,203,204,205,206,207,208,209,210,211,212,213,214,215,216,217,218,219,220,221,222,223,224,225,226,227,228,229,230,231,232,233,234,235,236,237].
