# Supplementary material for: Dendritic Cell Therapy in Immuno-Oncology: A Potentially Key Component of Anti-Cancer Immunotherapies
Source: Cancers (Basel). 2025 Dec 30;18(1):123. doi: 10.3390/cancers18010123 (PMC12785138; doi:10.3390/cancers18010123)
Supplement: Supplementary file 1 [file cancers-18-00123-s001.zip › cancers-4001084-supplementary.pdf]

Table S1. Summary of clinical trials investigating the use of dendritic cells in clinical settings reported with accessible study results to date.

Type Cellular Therapy	Target Antigen	Combination	Neoplasm	Study Type	Cohort Number	Clinical Observation	PFS/OS Data	Study ID or Title	References
Autologous plastic adherent monocyte-derived dendritic cells co-electroporated with autologous tumor total mRNA and synthetic CD40L mRNA	autologous tumor antigens	N/A	metastatic clear cell renal cell carcinoma (mRCC)	Phase II	21	Thirteen patients (62%) experienced clinical benefit (9 partial responses, 4 with stable disease); however, there were no complete responses in this group of intermediate and poor risk mRCC patients and enrollment was terminated early	Median PFS from registration was 11.2 months and the median OS from registration was 30.2 months.	NCT00678119	[157]
Autologous dendritic cells exposed to a human prostate adenocarcinoma cell line (LNCaP) killed by immunogenic modality (stapuldencel)	tumor antigens from LNCaP tumor cells	N/A	Metastatic Castration-Resistant Prostate Cancer	Phase III	1182	well-tolerated;<br>combined with docetaxel plus prednisone and continued as maintenance treatment did not extend OS in patients with mCRPC	N/A	NCT02111577	[106]
200 Gy-irradiated allogenic plastic adherent PBMC-derived dendritic cells fused with autologous tumor-derived cells	tumor antigens from autologous tumor cells	N/A	metastatic renal cell carcinoma (RCC)	Phase I/II	24	Vaccination resulted in anti-tumor immune responses in 10/21 evaluable patients as manifested by an increase in CD4 and/or CD8+ T cell expression of interferon-γ after ex vivo exposure to tumor lysate.	The median progression-free survival and overall survival was 18.1 and 116 weeks, respectively. At 1 year, progression-free survival and overall survival was 25% and 74%, respectively.	Phase I/II Study of Vaccination With Electrofused Allogeneic Dendritic Cells/Autologous Tumor-derived Cells in Patients With Stage IV Renal Cell Carcinoma	[158]
30 Gy-irradiated allogenic plastic	tumor antigens from	IL-2	metastatic renal cell cancer	Phase I/II	7	No objective response could be documented. However, while all patients had documented progress at	29% of the patients showed SD throughout the study with a mean time to	Allogeneic partially HLA-matched dendritic	[103]

adherent PBMC-derived dendritic cells	freezing/thawing-lysed autologous tumor cells irradiated with 25 KGy					study entry, 29% of the patients showed SD. In 3/7 patients, TH1-polarized immune responses against RCC-associated antigens were observed.	progression of 24.6 weeks (range 5 to 96 weeks)	cells pulsed with autologous tumor cell lysate as a vaccine in metastatic renal cell cancer; A clinical phase I/II study	
Allogeneic plastic adherent monocyte-derived dendritic cells	N/A	N/A	Renal cell carcinoma	Phase I	12	Treatment is safe<br>A massive infiltration of CD8+ T cells<br>No objective tumor response but regression of brain metastases<br>Improved median overall survival	Median mOS is not reached at 42.5 months	NCT01525017	[104]
Allogenic dendritic cells	allogeneic tumor antigens from 3 freezing/thawing cycles-lysed LNCaP and DU145 cell lines	N/A	hormone-refractory prostate cancer and metastatic renal cell carcinoma	Phase I/II	16	no evidence of significant toxicity;<br>increased expression of T helper type 1 interferon-gamma;<br>reduction in the level of prostate-specific antigen (PSA) in one patient, a reduction in PSA velocity in a further man and an increased PSA doubling time in six;<br>Two of five patients with renal cell carcinoma had stabilization of disease	N/A	Dendritic cell immunotherapy for urological cancers using cryopreserved allogeneic tumor lysate-pulsed cells: a phase I/II study	[105]
Allogenic plastic adherent PBMC-derived dendritic cells fused with autologous tumor cells	Autologous tumor antigens	N/A	metastatic renal cell carcinoma	Phase I/II	12	well-tolerated;<br>cytotoxicity of peripheral blood lymphocytes (PBLs) against renal cell carcinoma cells increased	N/A	Allogeneic dendritic cells fused with tumor cells: preclinical results and outcome of a clinical phase I/II trial in patients with metastatic renal cell carcinoma	[159]

Autologous CD14+ monocytes-derived dendritic cells	tumor antigens from OV-90 and SK-OV-3 cell line homogenates	N/A	epithelial ovarian cancer	Phase II	99	good safety profile; statistically significant improvement in PFS	Median PFS was 20.3, not reached, and 21.4 months in Groups A, B, and C, respectively. The HR (95% CI) for Group A versus Group C was 0.98 (0.48 to 2.00; $p = 0.9483$) and the HR for Group B versus Group C was 0.39 (0.16 to 0.96; $p = 0.0336$).	NCT02107937; EudraCT2010–021462–30	[160]
Autologous CD141+ conventional type 1 DCs are enriched in two subsequent selection steps using a immunomagnetic isolation. First, in a negative selection step, the product will be depleted of CD19+ B-cells, CD3+ T cells and CD14+ monocytes. Second, in a positive selection step, CD141+ DCs are selected.	tumor antigens from 5 freezing/thawing cycles-lysed autologous tumor cells and keyhole limpet hemocyanin	N/A	advanced epithelial ovarian cancer	Phase I/II	10	N/A	preparatory stage	NEODOC study NCT05773859; EUCT 2024–512353–24–01	[161]
Autologous conventional and plasmacytoid dendritic cells enriched by a 2-step immunomagnetic isolation. Negative selection consisted of depleting monocytes and B-cells using magnetic bead-coupled	Mucin-1 and Survivin	carboplatin/paclitaxel	Metastatic endometrial cancer	Phase I/II	7	Production of DC vaccines was successful in five out of seven patients;<br>All patients had at least one adverse event;<br>Antigen-specific responses could be demonstrated in two of the five patients	N/A	NCT04212377	[162]

CD14 and CD19 antibodies, respectively. Positive selection for pDCs was done using magnetic bead-coupled anti-BDCA4. cDC2s were first labeled with anti-BDCA1-biotin and subsequently selected with magnetic bead-coupled anti-biotin antibodies.								
autologous DCs	N/A	Temozolomide and radiation therapy	glioblastoma multiforme (GBM)	Phase II	76	Patients with favorable pre-existing anti-tumor characteristics lived longer under Audencel than Audencel patients without them. Pre-vaccination blood CD8+ T cell counts and ELISPOT Granzyme B production capacity in vitro upon tumor antigen exposure were significantly correlated with overall survival.	Progression-free survival at 12 months did not differ significantly between the control and vaccine groups (28.4% versus 24.5%). Median overall survival was similar with 18.3 months (vaccine: 564 days, versus control: 568 days)	NCT01213407; EudraCT2009–015979–27	[163]
Autologous dendritic cells	tumor antigens autologous tumor cells	carboplatin, gemcitabine or paclitaxel	Platinum-sensitive recurrent epithelial ovarian cancer	Phase II	132	DCVAC/OvCa combined with chemotherapy had a favorable safety profile in patients with platinum-sensitive ovarian cancer. DCVAC/OvCa did not improve PFS, but the exploratory analyses revealed OS prolongation and enhanced surrogate antigen-specific T cell activity.	Median PFS: 13.4 months (DCVAC/OvCa) vs 11.3 months (chemo alone); HR = 0.63 (95% CI: 0.41–0.97; p = 0.037). Median OS: 35.5 vs 29.9 months; HR = 0.67 (95% CI: 0.41–1.09; p = 0.11). 2-year OS: 71% vs 59%	NCT0210795	[164]
Autologous Dendritic Cells DCVax	autologous tumor lysate	N/A	Recurrent Glioblastoma	Phase III	331	Survival at 24 and 30 months after recurrence was 20.7% vs control 9.6% and 11.1% vs control 5.1%	Median OS was 19.3 (95% CI, 17.5–21.3) vs 16.5 (95% CI, 16.0–17.5) months of control;	NCT00045968	[110,111]

						Survival was improved in patients with methylated MGMT	rGBM median OS was 13.2 (95% CI, 9.7–16.8) months from relapse vs 7.8 (95% CI, 7.2–8.2) months		
autologous dendritic cells expressing full-length wild-type p53 gene	TP53	All-trans retinoic acid	Small cell lung cancer with TP53 mutation	Phase II	69	Although the DC vaccine failed to improve ORRs to the second-line chemotherapy, its safety profile and therapeutic immune potential remain	ORR: were 15.4% (95% CI 2.7–46.3) for placebo, 16.7% (95% CI 2.9–49.1) for vaccine alone, and 23.8% (95% CI 9.1–47.5) for vaccine with 150 mg/m^2 ATRA with no survival differences between arms	NCT03406715	[122]
Autologous Immunomagneticly-isolated CD34+ hematopoietic stem cell (HSC)-derived dendritic cells	tumor antigens from 5 freezing/thawing cycles-lysed autologous tumor cells	IL-2	Renal cell carcinoma and breast cancer	Phase I/II	10 (Six renal cell carcinoma and four breast cancer patients)	Clinical response was observed in one RCC patient as stable disease.	N/A	Combination therapy of renal cell carcinoma or breast cancer patients with dendritic cell vaccine and IL-2: results from a phase I/II trial	[165]
Autologous plastic adherent monocyte-derived dendritic cells	modified HLA-class I p53 peptides (Arm 1), additional Th tetanus toxoid peptide (Arm 2), or additional Th wild-type (wt) p53-specific peptide	N/A	head and neck cancer	Phase I	16	No grade II-IV adverse events were observed;<br>p53-specific T cell frequencies were increased postvaccination in 11 of 16 patients (69%), with IFN-gamma secretion detected in four of 16 patients. Treg frequencies were consistently decreased (p = 0.006) relative to pre-vaccination values.	N/A	UPCI 03–156; IRB# 0507062; NCT00798655	[166]

Autologous plastic adherent monocyte-derived dendritic cells	N/A	ipilimumab or pembrolizumab; cyclophosphamide	metastatic castration-resistant prostate cancer	Phase I	18	safe and well-tolerated; Results indicate anti-tumor activity through altered T cell receptor repertoires	33% durable (> 46 weeks) clinical benefit; median OS 40.7 months	NCT02423928; CryoIT	[167]
Autologous plastic adherent monocyte-derived dendritic cells	HLA-A2: WT1126–134 (RMFPNAPYL), WT1235–243 (CMTWNQMNL), gp100209–217 (IMDQVPFSV), HER2369–377 (KIFGSLAFL), MAGE-A3271–279 (FLWGPRALV); HLA-A24: WT1235–243M (CYTWNQMNL), WT1235–243 (CMTWNQMNL), HER263–71	N/A	High-grade glioma	Phase I	9	DC injections were well-tolerated except for transient liver dysfunction with grade II. Six patients showed positive immunological responses to peptides in an ELISPOT assay.	N/A	UMIN ID: 000000914	[168]

	(TYLPTNASL), MAGE-A1135–143 (NYKHCFPEI), MAGE-A3195–203 (IMPKAGLLI)								
Autologous plastic adherent monocyte-derived dendritic cells	tumor antigens from 6 freezing/thawing cycles-lysed autologous glioma stem cells irradiated with 100 Gy	Temozolomide and radiation therapy	glioblastoma	Phase II	57	Disease control and overall survival (OS) dropped suddenly once DC-ATA was completed. PFS was encouraging, and the data suggest that OS may be increased by extending vaccine treatment.	The median PFS of 10.7 months is 57.4% longer than the 6.8-month average of PFS medians from the adjuvant TMZ control arms of six randomized trials. The median OS of 16.0 months is 5.9% less than the 17.0-month median from the same control arms.	NCT03400917	[169]
Autologous plastic adherent monocyte-derived dendritic cells	tumor antigens from 5 freezing/thawing cycles-lysed autologous tumor cells irradiated with 25 KGy	Avelumab	MSS metastatic colorectal cancer	Phase I/II	19	Stimulation of immune system was observed in vitro but not clinically. The evaluation of basal RNA-seq noted significant changes between pre and post-therapy liver biopsies related to lipid metabolism and transport, inflammation and oxidative stress pathways.	Median PFS was 3.1 months [2.1–5.3 months] and overall survival was 12.2 months [3.2–23.2 months]	NCT03152565	[80]
Autologous plastic adherent monocyte-derived dendritic cells	tumor antigens from 5 freezing/thawing cycles-	N/A	Metastatic colorectal cancer	Phase II	52	ADC generates a tumour-specific immune response but not benefit on PFS and OS significantly	PFS was 2.7 months (95% CI: 2.3–3.2 months) with ADC + BSC vs. 2.3 months (95% CI: 2.1–2.5 months) with BSC OS was 6.2	NCT01413295	[170]

	lysed autologous tumor cells irradiated with 25 KGy						months (95% CI: 4.4–7.9 months) ADC + BSC vs. 4.7 months (95% CI: 2.3–7 months) with BSC Responder's OS: 7.3 months (95% CI: 5.2–9.4 months) p = 0.026		
Autologous plastic adherent monocyte-derived alphaDC1 dendritic cells	tumor antigens from UV B and γ-irradiation (20,000 Rads) apoptotic tumor cells co-cultured with DCs	INFα, rintatolimod, and celecoxib	Peritoneal Metastases	Phase II	46	well-tolerated;<br>PFS did not meet the predefined thresholds	Median progression-free survival (PFS) for appendiceal primaries was 50.4, 34.2, and 8.9 months for grade 1, 2, and 3 tumors, respectively, while median PFS for colorectal cancer was 20.5 and 8.9 months for moderately and poorly differentiated tumors, respectively	NCT02151448	[101]
Autologous plastic adherent monocyte-derived alpha-type-1 dendritic cells	5 glioma-specific synthetic peptides restricted to HLA A2 or A24 keyhole limpet hemocyanin	Temozolomide and radiation therapy	High-grade Glioma	Phase II	16	No severe side effects;<br>10/15 pts showed positive CTL responses	median PFS 11.0 month;<br>median OS 19.0 months	JRCT c040190103	[171]
Autologous plastic adherent monocyte-derived dendritic cells	N/A	radiotherapy	Soft tissue sarcoma	Phase II	18	No toxicities or serious adverse events.<br>Immunological responses<br>67% remain alive with no disease recurrence	PFS at two years is 83% and at four years is 67%;<br>median OS was 57 months	NCT00365872	[127]

Autologous plastic adherent monocyte-derived dendritic cells	HLA-A2: MART-1, gp100, tyrosinase, MAGE-A2, and MAGE-A3 and HLA-A24: gp100, tyrosinase, MAGE-A1, MAGE-A2 and MAGE-A3	N/A	metastatic melanoma	Phase II	24	DC ratio (lin-HLA-DR+) of the vaccine was 38.1+/-13.3% and the frequency of CD83+ DCs was 25.7+/-20.8%; DC processing-related parameters including injected DC numbers, DC ratio, and surface markers such as CD83 and CCR7 did not demonstrate any relationship to overall survival;	OS 13.6 M in vaccinate vs. 7.3 M in non-vaccinated; 21.9 M in high Immune response vs. 8.1 M in low	Dendritic cell-based vaccination in metastatic melanoma patients: phase II clinical trial	[172]
Autologous plastic adherent monocyte-derived dendritic cells co-cultured with immunogenically enhanced and 100 Gy irradiated autologous glioma cells	tumor antigens autologous tumor cells	N/A	Malignant glioma	Phase I/II	17	Dendritic cell treatment increased tumor shrinkage and increased concentration of tumor-infiltrating $CD8^+$ lymphocytes but may cause transient elevation of liver enzymes in the serum.	Median survival: 525 days vs. 380 days historically 5-year survival: 18.8% vs. 0% historically	EY-DOH-MD #0910072504	[99]
Autologous plastic adherent monocyte-derived dendritic cells electroporated co-electroporated with mRNA for CD40L, CD70, and caTLR4 and mRNA encoding one of four MAAs (MAGE-A3, MAGE-C2, tyrosinase, or gp100) linked to an	melanoma-associated antigen (MAA); either MAGE-A3, MAGE-C2, tyrosinase or gp100) (TriMixDC-MEL)	N/A	advanced melanoma	Phase I	15	Two patients achieved a complete response and two patients a partial response. All objective responders are progression-free after a follow-up of, respectively, 24+, 28+, 33+, and 34+ months.	N/A	NCT01066390	[173]

HLA class II targeting signal								
Autologous plastic adherent monocyte-derived HSP70 mRNA-electroporated dendritic cells	N/A	N/A	hepatocellular carcinoma	Phase I/II	45	No adverse events;	median OS was significantly longer in the DC group than in the control group (*p* = 0.044); 3-year OS rate was 96.3% vs 70.0% of control	UMIN000010691; IRB number: H24–40	[174]
Autologous plastic adherent PBMC or CD14+ magnetic beads-isolated monocyte-derived type-1-polarized dendritic cells	HLA-A2-presented tumor blood vessel antigens (DLK1, EphA2, HBB, NRP1, RGS5, TEM1)	immediate (arm B) or delayed (arm A) dasatinib	Melanoma	Phase II	16	Arm B (immediate dasatinib) outperformed those in Arm A (delayed dasatinib) for immune response rate (IRR; 66.7% vs 28.6%) and objective response rate (ORR) (66.7% vs 0%)	progression-free survival (median 7.87 vs 1.97 months; *p* = 0.063, arm B vs A); overall survival (median 15.45 vs 3.47 months; *p* = 0.0086, arm B vs A)	UPCI 12–048 NCT01876212	[126]
Autologous plastic adherent PBMC-derived dendritic cells	Co-cultured with UV irradiated apoptotic LNCaP prostate tumor cells	N/A	Prostate cancer	Phase I	24	induced delayed type hypersensitivity (DTH) responses and CD4+ and CD8+ T cell proliferation; statistically significant increase in T cell proliferation responses to prostate tumor cells in vitro (*p*=0.002); decrease in prostate specific antigen (PSA) slope (*p*=0.016); two-fold increase in PSA doubling time (*p*=0.003)	N/A	NCT00289341	[106]
Autologous plastic adherent PBMC-derived dendritic cells	Yeast cell wall-linked autologous melanoma tumor lysate	GM-CSF prior immunotherapy	Melanoma	Phase IIB	187	Improved disease-free and overall survival Both TLPO and TLPLDC show clinical benefit No difference in DFS and OS for TLPO and TLPLDC	DFS was 27.2% (placebo), 55.4% (TLPLDC), 22.9% (TLPLDC + G), and 60.9% (TLPO) (*p* < 0.001). OS was 62.5% (placebo), 93.6% (TLPLDC), 57.7%	NCT02301611	[107]

							(TLPLDC + G), and 94.6% (TLPO) ($p = 0.002$)		
Autologous plastic adherent PBMC-derived dendritic cells	25 KGy-irradiated autologous tumors cells lysed by five freezing/thawing cycles	Avelumab (anti-PD-L1)	Mismatch repair-proficient (MSS) metastatic colorectal cancer	Phase I/II	28	combination of Avelumab plus ADC vaccine is safe and well-tolerated; Modest clinical activity	median OS of 12.2 months	NCT03152565	[80]
Autologous plastic adherent PBMC-derived dendritic cells	PANVAC-V and PANVAC-F recombinant vaccinia virus containing CEA, MUC1, and 3 costimulatory molecules B7.1, ICAM-1, and LFA-3	GM-CSF	Metastatic colorectal cancer	Phase II	74	Recurrence-free survival at 2 years was similar (47% and 55% for DC/PANVAC and PANVAC/GM-CSF, respectively);<br>OS for vaccinated patients combined compared with contemporary, unvaccinated controls	Median RFS (95% CI) was 21.9 (16.9–38.8) and 25.7 (20.0–37.2), months, respectively;<br>Median OS (95% CI) was not reached and 44.1 (36.2–63.4), months	NCT00103142	[120]
Autologous plastic adherent PBMC-derived dendritic cells	TP53	All-trans retinoic acid	Small cell lung cancer	Phase II	78	Vaccine was safe;<br>positive immune responses in DC only patients (3/15, 20.0%; 95% confidence interval [CI], 5.3–48.6%)<br>positive immune responses in DC + ATRA patients (10/23, 43.3%; 95% CI 23.9–65.1%)<br>but failed to improve overall response	N/A	NCT00617409	[122]
Autologous plastic adherent PBMC-derived dendritic cells	TP53 via transduction by serotype	Indoximod	Solid tumors and	Phase I	39	No dose-limiting toxicities;<br>Monotherapy of both DC vaccine and chemotherapy showed no significant	median PFS was 13.3 weeks (95% CI, 12.97–21.85)	NCT01042535	[123]

	5 adenoviral vector, containing the wild-type p53		invasive breast cancer			objective responses; no statistically significant difference in progression-free survival or overall survival between the immunologic responders and nonresponding ones Putative chemo-sensitization effect	median OS was 20.71 weeks (95% CI, 25.75–46.15)		
Autologous plastic adherent PBMC-derived dendritic cells	120 KGy-irradiated autologous tumors cells lysed by five freezing/thawing cycles	N/A	Solid extra-cranial tumors	Phase I	24	mDC1 is safe induction of IL-12	N/A	Phase I study of tumor Ag-loaded IL-12 secreting semi-mature DC for the treatment of pediatric cancer	[136]
Autologous plastic adherent PBMC-derived dendritic cells	DCs fused with multiple myeloma cells	GM-CSF and lenalidomide	Multiple myeloma	Phase II	203	Significant increase in circulating multiple myeloma–reactive lymphocytes No statistically significant increase in clinical response rates at 1-year posttransplant	N/A	NCT02728102	[137,138]
Autologous plastic adherent PBMC-derived dendritic cells	autologous tumors cells lysed by undisclosed number of freezing/thawing cycles, linked to yeast cell wall particles	N/A	Solid tumors	Phase I/II	31	vaccine is safe 39% demonstrated various clinical responses including complete and partial responses as well as stable disease response	median PFS 22.5 months	ISRCTN81339386	[140]
Autologous plastic adherent PBMC-derived dendritic cells	gp100- and tyrosinase- and tetanus helper peptides	either GM-CSF or Montanide	Melanoma	Phase II	26	objective clinical response rates in the GM-CSF arm and DC arm were 15% (95% CI, 2% to 45%) and 8% (95% CI, 1% to 36%), respectively	N/A	Clinical and immunologic results of a randomized phase II trial of	[150]

		ISA-51 adjuvant				peptide-specific immune responses in 75% to 80% of patients		vaccination using four melanoma peptides either administered in granulocyte-macrophage colony-stimulating factor in adjuvant or pulsed on dendritic cells	
Autologous plastic adherent PBMC-derived dendritic cells	survivin, hTERT, and p53-derived peptides (HLA-A2(+)) or tumor lysate (HLA-A2(-))	IL-2, Cyclophosphamide, and Celecoxib	Metastatic melanoma	Phase II	28	The treatment was safe and tolerable	median PFS was 4.5 months<br>median OS was 9.4 months	NCT00197912	[175]
Autologous plastic adherent PBMC-derived dendritic cells	Tumor antigens from whole tumor lysate	before radiotherapy and was continued during adjuvant chemotherapy	newly diagnosed patients GBM		32	No severe adverse effects	Median PFS was 12.7 months (CI 95% 7–16)<br>median OS was 23.4 months (95% CI 16–33.1)	2009–009879–35 and NCT01006044	[176]
Autologous plastic adherent PBMC-derived dendritic cells	α-galactosyl ceramide		advanced non-small cell lung or recurrent	Phase I	11	No severe adverse events<br>dramatic increase in peripheral blood Valpha24 NKT<br>No patient was found to meet the criteria for partial or complete	N/A	no. 333;<br>A phase I study of alpha-galactosyl ceramide (KRN7000)-pulsed	[177]

			lung cancer			responses, whereas two cases in the level 3 group remained unchanged for more than a year with good quality of life.		dendritic cells in patients with advanced and recurrent non-small cell lung cancer	
Autologous plastic adherent PBMC-derived dendritic cells	tumor lysate (TL), keyhole limpet hemocyanin	tumor necrosis factor-alpha (TNF-alpha)	unresectable primary liver cancer	Phase I	10	Immunization was well-tolerated DC vaccination induced delayed-type hypersensitivity (DTH) against KLH in seven out of ten patients. In one patient, one of the two liver tumors (tumor in segment 7, 13 mm in diameter) decreased in size to 7 mm and showed necrotic change on computed tomography examination after eight immunizations. In two patients, serum levels of tumor markers decreased after vaccination.	N/A	A phase I study of autologous dendritic cell-based immunotherapy for patients with unresectable primary liver cancer	[178]
Autologous plastic adherent PBMC-derived dendritic cells	wild-type peptides, gp100(aa154–167), gp100(aa280–288), and tyrosinase(aa369–376), keyhole limpet hemocyanin	daclizumab, a humanized antibody against the interleukin-2 (IL-2) receptor alpha-chain (CD25)	metastatic melanoma patients	Phase I/II	30	Daclizumab efficiently depleted all CD25(high) Treg cells for 4 weeks, not enhance the efficacy of the dendritic cell	N/A	Dendritic Cell Vaccination in Combination with Anti-CD25 Monoclonal Antibody Treatment: A Phase I/II Study in Metastatic Melanoma Patients	[179]

Autologous plastic adherent PBMC-derived dendritic cells	tumor antigens from autologous UV B-irradiated myeloma cells		relapsed or refractory multiple myeloma	Phase I	9	therapy was well-tolerated immunological response (77.8%)	clinical benefit rate was 66.7%	NCT02248402	[180]
Autologous plastic adherent PBMC-derived dendritic cells	tumor antigens from 5 freezing/thawing cycles-lysed autologous tumor cells and keyhole limpet hemocyanin		metastatic renal cell carcinoma	Phase I/II	9	was well-tolerated without severe side effects	median PFS was 5.2 months median OS was 29 months	0412-CR02–0704–0001	[181]
Autologous plastic adherent PBMC-derived dendritic cells	MAGE-A1, MAGE-A3 and NY-ESO-1 peptide	decitabine	relapsed or therapy-refractory neuroblastoma and sarcoma	Phase I	9	well-tolerated;<br>1/10 patients who had a complete response	N/A	NCT01241162	[182]
Autologous plastic adherent PBMC-derived dendritic cells	tumor antigens from 3 freezing/thawing cycles-lysed autologous tumor cells	interleukin-2	thyroid cancer	Phase I	6	No adverse events	N/A	Results of a phase I clinical study using dendritic cell vaccinations for thyroid cancer	[183]

Autologous plastic adherent PBMC-derived dendritic cells	MART-1 (aa27–35), tyrosinase (aa368–376) (370D) and gp100 (aa209–217) (210M) peptides	N/A	metastatic melanoma	Phase I	16	Toxicity was minimal	N/A	Phase I trial of intravenous peptide-pulsed dendritic cells in patients with metastatic melanoma	[90]
Autologous plastic adherent PBMC-derived dendritic cells	MUC1 (GVTSAPD TRPAPGST APPAH)5	N/A	pancreatic and biliary tumors	Phase I/II	12	well-tolerated and no toxicity was observed; increases in activity of all CD8 and CD4 T cells	median survival is 26 months (range 13–69 months)	Investigational New Drug (IND) # 10467	[184]
Autologous plastic adherent PBMC-derived dendritic cells	mannan-conjugated fusion protein of MUC1 including three copies of the VNTR motif (PDTRPAP GSTAPPAH GVTSA) and glutathione-S-transferase	N/A	adenocarci noma	Phase I	10	strong T cell IFNγ responses	PFS over 3 years in 20% patients	Mannan-MUC1-pulsed dendritic cell immunotherapy: a phase I trial in patients with adenocarcinoma	[185]
Autologous plastic adherent PBMC-derived dendritic cells	tumor antigens from 5 freezing/tha wing cycles-lysed	N/A	metastatic renal cell carcinoma	Phase I/II	15	well-tolerated	N/A	Therapeutic vaccination against metastatic renal cell carcinoma by autologous dendritic cells:	[186]

	autologous tumor cells							preclinical results and outcome of a first clinical phase I/II trial	
Autologous plastic adherent PBMC-derived dendritic cells	hTERT-peptides HLA-A*02: ILAKFLHWL, HLA-A*03: KLFGVLRLK, HLA-A*24: VYAETKHFL	IL-2	metastatic renal cell carcinoma	Phase I/II	10	well-tolerated; significant increase in cytotoxic activity of effector cells from all responders	N/A	Telomerase-pulsed dendritic cells: preclinical results and outcome of a clinical phase I/II trial in patients with metastatic renal cell carcinoma Approval of local ethics committees (AZ 110/02)	[187]
Autologous plastic adherent PBMC-derived dendritic cells	WT1, Her2, and CEA, MUC1 long peptide (30-mer), CA-125, and tumor antigens from 5 freezing/thawing cycles-lysed autologous tumor cells	gemcitabine	pancreatic cancer	Phase I	10	well-tolerated	N/A	UMIN-000004855	[188]
Autologous plastic adherent PBMC-derived dendritic cells	mannosylated mucin 1 protein	N/A	advanced epithelial ovarian cancer	Phase I/II	28	no Grade 3 or 4 toxicities; 5/24 pts > 25% CA125 reduction	N/A	A phase 2, single-arm study of an autologous dendritic cell treatment against	[189]

								mucin 1 in patients with advanced epithelial ovarian cancer	
Autologous plastic adherent PBMC-derived dendritic cells	HLA-A0201-specific prostate-specific membrane antigen (PSMA) peptides	N/A	prostate cancer	Phase I	51	No significant toxicity was observed; Cellular response against PSM-P1 and -P2 was observed in HLA-A2+ patients;	N/A	Phase I clinical trial: T cell therapy for prostate cancer using autologous dendritic cells pulsed with HLA-A0201-specific peptides from prostate-specific membrane antigen	[190]
Autologous plastic adherent PBMC-derived dendritic cells	HLA-A*24:02-restricted WT1 (235–243: CYTWNQMNL) and HLA-A*02:01/02:06-restricted WT1 peptide (126–134: RMFPNAPYL); MUC1 long peptide TRPAPGSTAPPAHGVTSAPDTRPAPGSTAP was used for	tegafur-gimeracil-oteracil potassium; gemcitabine	Pancreatic Ductal Adenocarcinoma	Phase I/II	10	no Grade 2 or higher CTCAE v5.0 toxicities were found; WT1-specific CTLs were detected; tendency for induction of WT1-specific CTL after DC vaccination in cases with a higher degree of infiltrations of CD3+, CD4+, or CD8+ MNCs	OS was 18.5–72.8 months (median 46.4 months); PFS 12.5–72.8 months (median 17.7 months); OS and RFS at 3-years were 77.8% (95%CI = 0.37–0.94) and 35.0% (95%CI = 0.09–0.64), OS and RFS at 5-year were 19.4% (95%CI = 0.01–0.55) and 23.3% (95%CI = 0.04–0.53)	Dendritic Cell-based Immunotherapy Pulsed With Wilms Tumor 1 Peptide and Mucin 1 as an Adjuvant Therapy for Pancreatic Ductal Adenocarcinoma After Curative Resection: A Phase I/IIa Clinical Trial approval number: 12102231	[191]

	all HLA-A types								
Autologous plastic adherent PBMC-derived dendritic cells	tumor antigens from 3 freezing/thawing cycles-lysed autologous tumor cells	tumor necrosis factor-alpha	malignant melanoma	Phase I	10	minimal adverse events; In vitro, melanoma patient-derived dendritic cells (DCs) showed reduced cell surface expression of CD1a antigen on iDCs and reduced CD86 and HLA-DR expression on mDCs	Median survival was 240 days (range 31–735 days)	Results of a phase I clinical study using autologous tumor lysate-pulsed monocyte-derived mature dendritic cell vaccinations for stage IV malignant melanoma patients combined with low dose interleukin-2	[89]
Autologous plastic adherent PBMC-derived dendritic cells	tumor antigens from irradiated autologous tumor cells	interleukin-2	melanoma cell	Phase I/II	19	minimal side-effects; S-100B levels prior to the commencement of treatment correlated with objective clinical response; Three of the 12 patients who completed priming have durable complete responses (average duration 35 months+), three had partial responses, and the remaining six had progressive disease (WHO criteria).	N/A	Durable complete clinical responses in a phase I/II trial using an autologous melanoma cell/dendritic cell vaccine	[192]
Autologous plastic adherent PBMC-derived dendritic cells	Wilms' tumor 1 peptide and OK-432 adjuvant	conventional chemotherapy	head and neck squamous cell carcinoma	Phase I/II	11	well-tolerated and no serious adverse events	Median PFS was 6.4 months (1.9–2.0); Median OS 12.1 months (4.9–6.0)	UMIN 000027279	[193]
Autologous plastic adherent PBMC-derived dendritic cells	tumor antigens from 3 freezing/tha	interleukin-2	metastatic renal cell carcinoma	Phase I	12	DC phenotype was CD14(low), CD86(high), CD40(high), CD80(low), and CD83(low); proliferative responses against TuLy	Objective clinical responses were not observed	Vaccination of Patients With Metastatic Renal Cell Carcinoma	[194]

	wing cycles-lysed autologous tumor cells					were not detected, and humoral responses against TuLy or KLH were absent		with Autologous Dendritic Cells Pulsed With Autologous Tumor Antigens in Combination With Interleukin-2: A Phase 1 Study	
Autologous plastic adherent PBMC-derived dendritic cells	HLA-A2 restricted to WT1 peptide (RMFPNAPYL), CEA peptide (YLSGANLNL), MAGE-1 peptide (KVAELVHFL), and HER-2 peptide (KIFGSLAFL)	N/A	non-small cell lung cancer	Phase I	5	improvement in the specific immune response;<br>Three patients had a longer time survival than expect	N/A	ISRCTN45563569	[156]
Autologous plastic adherent PBMC-derived dendritic cells	HLA-A1 restricted, MAGE1(161) EADPTGHSY, AIM-2(14) RSDSGQQARY, and HLA-A2	N/A	newly diagnosed glioblastoma	Phase I	21	Expression of four ICT-107 targeted antigens in the pre-vaccine tumors correlated with prolonged overall survival and PFS	Median PFS was 16.9 months;<br>median OS was 38.4 months	Phase I trial of a multi-epitope-pulsed dendritic cell vaccine for patients with newly diagnosed glioblastoma	[88]

	restricted, TRP-2(180) SVYDFFVWL, gp100(210M) IMDQVPFSV, HER2(773) VMAGVGSPYV and IL13Rα2(345) WLPFGFILI							
Autologous plastic adherent PBMC-derived dendritic cells	tumor antigens from UV-irradiated LNCaP cell line	cyclophosphamide; imiquimod;	metastatic, castration-resistant prostate	Phase I/II	25	No serious adverse events; significant decrease in Tregs in the peripheral blood; long-term administration of DCVAC/PCa led to the induction and maintenance of PSA specific T cells;	median OS was 19 months, whereas the predicted median OS was 11.8	EudraCT 2009–017295–24	[83]
Autologous plastic adherent PBMC-derived dendritic cells	tumor antigens from lysates of M44, COLO829 and SK-MEL28 melanoma cell lines or melanoma tumor antigens listed	N/A	advanced melanoma	Phase II	33	significant immunogenicity and anti-tumor activity	prospectively defined tumor growth control rate was 27% (90% confidence interval of 13–46%)	NCT00107159	[195]

Autologous plastic adherent PBMC-derived dendritic cells	tumor antigens from autologous tumor homogenate keyhole limpet hemocyanin	Temozolomide and radiation therapy	glioblastoma	Phase II	9	well-tolerated	median PFS from leukapheresis was 11.3 months and 12.2 months	Eudract number: 2020–003755–15	[81]
Autologous plastic adherent PBMC-derived dendritic cells	CEA. 652(9)	N/A	colorectal cancer	Phase I/II	10	well-tolerated; In the SD patient, natural killer (NK) cell frequency and cytolytic activity were increased. In the same patient, the frequency of CEA-specific cytotoxic T cells (CTLs) increased stepwise with repetitive vaccinations; however, most of the CTLs exhibited central memory phenotype. In those with PD, NK cells proliferated well regardless of failure of response, whereas CTLs failed to do so.	N/A	UMIN000000743	[196]
Autologous plastic adherent PBMC-derived dendritic cells	human papillomavirus type 16 (HPV16) or HPV18 (HPV16/18) E7 antigen	N/A	cervical cancer	Phase I	10	well-tolerated; All patients developed CD4(+) T cell and antibody responses; 8 out of 10 patients demonstrated levels of E7-specific CD8(+) T cell counts;	N/A	BB-IND-11307	[197]
Autologous plastic adherent PBMC-derived dendritic cells	alpha-fetoprotein, glypican-3 and MAGE-1 recombinant	imiquimod	hepatocellular carcinoma	Phase I/II	5	all 5 patients demonstrated strong T cell responses against HCC antigens; no tumor response was observed in the other 4 patients;	N/A	Institutional Review Board of Ehime University Hospital Approval ID #0809003	[86]

	fusion proteins								
Autologous plastic adherent PBMC-derived dendritic cells	tumor antigens from 5 freezing/thawing cycles-lysed autologous tumor cells	N/A	non-small cell lung cancer	Phase I	15	well-tolerated; 9 revealed no change in the immunologic response; small sample size and universally poor outcome precluded meaningful statistical assessment	N/A	Phase I study of autologous dendritic cell tumor vaccine in patients with non-small cell lung cancer	[86]
Autologous plastic adherent PBMC-derived dendritic cells	tumor antigens from freezing/thawing-lysed, irradiated autologous tumor cells	subcutaneous injection of GM-CSF	melanoma	Phase I/II	63	43 TLPO and 20 TLPLDC pts; no differences in DFS or OS in resected Stage III/IV melanoma patients receiving adjuvant TLPO versus TLPLDC vaccines	median follow-up of 20.5 months, the DFS (60.8% vs. 58.7%, $p = 0.714$) and OS (94.6% vs. 93.8%, $p = 0.966$) were equivalent between the TLPO and TLPLDC groups, respectively	NCT02301611	[198]
Autologous plastic adherent PBMC-derived dendritic cells	tumor antigens from freezing/thawing-lysed, irradiated autologous tumor cells	N/A	malignant astrocytoma	Phase I	13	well-tolerated	N/A	Results of a phase I dendritic cell vaccine trial for malignant astrocytoma: potential interaction with adjuvant chemotherapy	[199]
Autologous plastic adherent PBMC-derived dendritic cells	tumor antigens from 5 freezing/thawing cycles-lysed autologous tumor cells	N/A	recurrent glioma	Phase I/II	24	All patients were resistant to the standard maximum therapy; well-tolerated; 1 patient with partial response, 3 patients with minor response, 10 patients with stable disease, and 10 patients with progressive disease; The patients whose dendritic cells	median overall survival time was 480 days (range, 63–1466 days) whereas in the control group, median overall survival time was 400 days (range, 136–814 days); overall survival was 23.5%	Clinical evaluation of dendritic cell vaccination for patients with recurrent glioma: results of a clinical phase I/II trial	[200]

						were matured with OK-432 had longer survival times than the dendritic cells from patients without OK-432 maturation;	at 2 years. In the control group the percentage of overall survival was 3.7% at 2 years		
Autologous plastic adherent PBMC-derived dendritic cells	HLA-A*24:02-restricted WT1 (235–243:CYTWNQMNL), HLA-A*02:01/02:06-restricted WT1 (126–134:RMFPNAPYL), and HLA class II-restricted peptides (332–347: KRYFKLSHLQMHSRKH)	N/A	Pancreatic Cancer	Phase I	8	No serious side-effects were observed	OS at 2 years after the operation was 62.5 ± 17.1% (95% confidence interval = 22.9–86.1%)	WT1-pulsed Dendritic Cell Vaccine Combined with Chemotherapy for Resected Pancreatic Cancer in a Phase I Study	[201]
Autologous plastic adherent PBMC-derived dendritic cells	tumor antigens from 5 freezing/thawing cycles-lysed autologous glioma stem cells irradiated with 6 cGy	N/A	glioblastoma:	Phase II	43	DCV treatment significantly prolonged OS; Among IDH1(wild type (WT)) TERT(MT) patients, DCV treatment significantly prolonged OS ($p < 0.01$) and PFS ($p = 0.03$) and increased plasma levels of cytokines CCL22 and IFN-gamma; Patients with low B7-H4 expression showed significantly prolonged OS ($p = 0.02$)	DCV treatment showed prolonged overall survival (OS) (median OS 13.7 months vs 10.7 months, $p = 0.05$), but not progression-free survival (PFS) (median PFS 7.7 months vs 6.9 months, $p = 0.75$); when the analysis was conducted using a multivariate Cox	NCT 01567202	[202]

							proportional hazards model adjusted for IDH1 and TERT promoter MT, primary vs recurrent GBM and B7-H4 expression, the DCV treatment group showed more significantly prolonged OS (p = 0.02; HR 2.5; 95% confidence interval [CI] 1.15–5.45) and a non-significant trend of improvement of PFS (p = 0.37; HR 1.37; 95% CI 0.68–2.74)		
Autologous plastic adherent PBMC-derived dendritic cells	idiotype of the myeloma immunoglobulin	N/A	multiple myeloma	Phase II	24	lower cumulative incidence of progression in the vaccinated group at 12 months	N/A	Efficacy and safety of Id-protein-loaded dendritic cell vaccine in patients with multiple myeloma--phase II study results	[203]
Autologous plastic adherent PBMC-derived dendritic cells	tumor antigens from 5 freezing/thawing cycles-lysed HHP-treated H522 and H520 cells	N/A	non-small cell lung cancer	Phase I/II	45	DCVAC/LuCa and chemotherapy (Group A); 29 (25.9%) to receive DCVAC/LuCa, chemotherapy and immune enhancers (Group B); and 38 (33.9%) to receive chemotherapy only (Group C); at 5 years six patients in the DCVAC/LuCa groups (4 in Group A and 2 in Group B) were alive; no patients were alive in the control group	median PFS in the mITT population was 1.1 months longer in Group A than in Group C (6.7 vs. 5.6 months) (p = 0.0334, unstratified log-rank test; HR = 0.58 [95% CI: 0.35–0.97]; median OS was 4.3 months longer in Group A than in Group C (16.4 vs. 12.1 months). This improvement was	NCT02470468	[204]

						statistically significant (p = 0.0316, unstratified log-rank test; HR = 0.55 [95% CI: 0.32–0.96]		
Autologous plastic adherent PBMC-derived dendritic cells	yeast cell wall particles-linked tumor antigens from freezing/thawing cycles-lysed autologous melanoma cells	N/A	resected stage III/IV melanoma	Phase IIB	144	Patients receiving TLPLDC vaccine without G-CSF had improved OS and DFS. Direct DC harvest without G-CSF had higher expression of genes linked to DC maturation.	36-months DFS:<br>TLPLDC: 55.8%<br>G-CSF + TLPLDC: 24.4%<br>Placebo: 30.0%<br><br>36 months OS:<br>TLPLDC: 94.2%<br>G-CSF + TLPLDC: 69.8%<br>Placebo: 70.9%	Divergent clinical outcomes in a phase 2B trial of the TLPLDC vaccine in preventing melanoma recurrence and the impact of dendritic cell collection methodology: a randomized clinical trial.	[205]
Autologous plastic adherent PBMC-derived dendritic cells	tumor antigens from 6 freezing/thawing cycles-lysed autologous glioma stem cells irradiated with 60 Gy	Radio-chemotherapy	high-grade glioma	Phase I/II	77	Full integration of autologous DC-based tumor vaccination into standard postoperative radio-chemotherapy for newly diagnosed glioblastoma seems safe and possibly beneficial.	The 6mo-PFS was 70.1% from inclusion. Median OS was 18.3 months.	EudraCT 2006–002881–20	[206]
Autologous plastic adherent PBMC-derived dendritic cells	HLA-matched survivin/telomerase peptide; Allogenic tumor	IL-2	metastatic renal cell carcinoma	Phase I/II	27	None of the patients had an objective response but 13/27 patients obtained disease stabilization (SD) for more than 8 weeks. An antigen-specific immune response was demonstrated in 6/6 patients tested	At a median follow-up time of 25.7 months (range: 20.8 to 34.9) the median overall survival was 16.6 months and the median progression-free survival	NCT00197860	[207]

	antigens from freezing/thawing cycles-lysed RCC cell of A-498, Caki-1, and Caki-2 cell lines irradiated with 200 Gy						was 2.7 months (range: 1 to 22.3).		
Autologous plastic adherent PBMC-derived dendritic cells	N/A	IL-2	resected stage III/IV melanoma.	Phase II	18	Pts receiving DC vaccination showed a better relapse-free survival compared to the observational cohort. Immune response data suggested increased immune modulation in vaccinated patients	median RFS: 6.6 months in DC vaccination cohort and 5.2 months in observational cohort.	NCT02718391; EudraCT no. 2014–005123–27	[208]
Autologous plastic adherent PBMC-derived dendritic cells	tumor antigens from acid-eluted tumor-associated surface proteins enriched for MHC class I peptides of autologous tumor cultures	resiquimod or poly-ICLC	Grade III-IV malignant gliomas	Phase II	23	PD-1 expression increases on CD4+T-cells, while CD38 and CD39 expression are reduced on CD8+T cells, alongside an increase in monocytes. Poly-ICLC treatment amplifies the induction of interferon-induced genes in monocytes and T lymphocytes.	Median progression-free survival (PFS) was 8.1 months; and median overall survival (OS) was 26.6 months.	NCT01204684	[209]
Autologous plastic adherent PBMC-derived dendritic cells	tumor antigens from autologous	Temozolomide	Glioblastoma	Phase II	9	Two patients had progressed within three months after leukapheresis, but none had experienced DCvax-related G3–4 toxicities. One patient	The median PFS from leukapheresis was 11.3 months and 12.2 months	EudraCT: 2020–003755–15	[81]

	tumor cell lysate					experienced a positive DTH skin test towards autologous tumor homogenate.	from surgery. Study still in progress.		
Autologous plastic adherent PBMC-derived dendritic cells	tumor antigens from UV-B-irradiated LNCaP tumor cells	cyclophosphamide	prostate cancer	Phase I/II	27	Long-term immunotherapy of prostate cancer patients experiencing early signs of PSA recurrence using DCVAC/PCa was safe, induced an immune response and led to the significant prolongation of PSA doubling time.	The median PSADT in all treated patients increased from 5.67 months prior to immunotherapy to 18.85 months after 12 doses.	EudraCT 2009–017259–91	[210]
Autologous plastic adherent PBMC-derived dendritic cells	idiotype of the myeloma immunoglobulin	N/A	multiple myeloma	Phase II	11	Immune responses measured by ELISpot were noted in 3/11 (27%) and DTH skin test for Id-protein was positive in 8/11 (73%) of patients.	During the follow-up with a median of 33.1 months (range: 11-43 months), the disease remained stable in 7/11 (64%) of patients.	Results of a Phase II clinical trial with Id-protein-loaded dendritic cell vaccine in multiple myeloma: encouraging or discouraging?	[211]
Autologous plastic adherent PBMC-derived dendritic cells	mannan-linked recombinant human fusion protein of mucin 1 GVTSAPDTRPAPGSTAPPAH and glutathione S-transferase	N/A	epithelial ovarian cancer (EOC)	Phase II	56	A variable but measurable mucin 1 T cell-specific response was induced in all CVac-treated and some standard of care (SOC) patients.	Progression free survival (PFS) was not significantly longer in the treated group compared to SOC group (13 vs. 9 months). OS for CR2 patients at 42 months of follow-up showed a difference of 26 months for SOC vs. > 42 months for CVac-treated (as median OS had not been reached).	NCT01068509	[121]
Autologous plastic adherent PBMC-derived dendritic cells	Yeast cell wall-linked autologous	N/A	Stage III/IV melanoma	Phase IIb	144	Vaccine was well-tolerated. Better immune response observed: increased tumor-infiltrating CD8+ lymphocytes. improved benefit in patients	2-year DFS: 62.9% (TLPLDC) vs. 37.2% (placebo).2-year OS: 86.4% (TLPLDC) vs. 75.1%	Multi-institutional, prospective, randomized, double-blind,	[212]

	melanoma tumor lysate					completing the full vaccine series, improved benefit in patients completing the full vaccine series	(placebo). Hazard ratio for recurrence: 0.45 (95% CI, 0.23–0.88).	placebo-controlled phase IIb trial of the tumor lysate, particle-loaded, dendritic cell (TLPLDC) vaccine to prevent recurrence in high-risk melanoma patients: A subgroup analysis	
Autologous plastic adherent PBMC-derived dendritic cells co-cultured with 20,000 cGy irradiated melanoma cells	tumor antigens from five freeze-thaw cycles-lysed 20,000 cGy irradiated melanoma cells	N/A	metastatic melanoma	Phase I	15	5 of 15 patients achieved delayed type hypersensitivity (DTH) responses and 6 of 15 had positive IFN-gamma production.	Survival from enrollment date ranged from 5–38 months with a mean of 21.2 months. On average, SD patients were stable for 5 months before disease progression.	UPCI #01–171	[102]
Autologous plastic adherent PBMC-derived dendritic cells co-cultured with immunogenically enhanced and 100 Gy irradiated autologous glioma cells	tumor antigens autologous tumor cells	N/A	Glioblastoma multiforme (WHO grade IV), newly diagnosed	Phase II	34	Few adverse effects; transient abnormal liver function and mild lymphopenia, improved quality of life, higher surviving fraction with vaccine but not with radiosurgery	OS: 31.9 months (vaccine) vs. 15.0 months (control), $p < 0.002$. PFS: 8.5 months (vaccine) vs. 8.0 months (control), $p = 0.075$.1-year survival: 88.9% (vaccine) vs. 75.0% (control). 2-year survival: 44.4% (vaccine) vs. 18.8% (control), $p = 0.035$.3-year survival: 16.7% (vaccine) vs. 0% (control), $p = 0.014$. Mortality: 0.23 (95% CI 0.07–0.77), $p = 0.017$.	Adjuvant Immunotherapy with Whole-Cell Lysate Dendritic Cells Vaccine for Glioblastoma Multiforme: A Phase II Clinical Trial	[100]

Autologous plastic adherent PBMC-derived dendritic cells electroporated with WT1 mRNA	WT1	N/A	Advanced uterine tumors (endometrial carcinoma and leiomyosarcoma), pre-treated	Phase I/II	6	WT1-specific CD8+ T cell responses detected in 2/4 HLA-A2+ patients; transient molecular or radiologic tumor regression observed in 3/4 HLA-A2+ cases. No immune or clinical response in HLA-A2– patients.	one stage IV serous endometrial carcinoma patient had PFS 6 mo, survival 36 mo; another stage III serous EC patient had PFS 3 mo, survival 43 mo; stage I LMS patients had PFS 2 mo (OS 22 mo) and PFS 0 mo (OS 20 mo)	NCT02107950	[213]
Autologous plastic adherent PBMC-derived dendritic cells fused with autologous myeloma cells	tumor antigens autologous tumor cells	GM-CSF and lenalidomide	Newly diagnosed multiple myeloma undergoing single autologous HCT	Phase II	203	vaccination with lenalidomide did not result in a statistically significant increase in CR rates at 1-year posttransplant but was associated with a significant increase in circulating multiple myeloma–reactive lymphocytes indicative of tumor-specific immunity, single-cell transcriptomics revealed clonotypic expansion of activated CD8 cells and shared dominant clonotypes between patients at 1-year posttransplant.	1 year CR were 52.9% (vaccine) and 50% (control; $p = 0.37$, 80% CI 44.5%, 61.3%, and 41.6%, 58.4%), rates of VGPR or better were 85.3% (vaccine) and 77.8% (control; $p = 0.2$). Conversion to CR at 1 year was 34.8% (vaccine) and 27.3% (control; $p = 0.4$) Vaccination induced a statistically significant expansion of multiple myeloma–reactive T cells at 1 year compared with before vaccination ($p = 0.024$)	BMT CTN 1401	[138]
Autologous plastic adherent PBMC-derived dendritic cells transduced with an adenoviral (Ad) vector expressing the CCL21	N/A	N/A	advanced non-small cell lung carcinoma	Phase I	16	6 of 16 patients had systemic responses against tumor-associated antigens; induction of systemic tumor antigen-specific immune responses; enhanced tumor CD8(+) T cell infiltration; increased tumor PD-L1 expression	Median survival was 3.9 months	NCT01574222	[214]

Autologous plastic adherent PBMC-derived dendritic cells transduced with an adenovirus encoding a truncated LMP1 (ΔLMP1) and the full-length LMP2 (Ad-ΔLMP1-LMP2)	truncated LMP1 (ΔLMP1) and the full-length LMP2 (Ad-ΔLMP1-LMP2)	N/A	Metastatic Epstein–Barr virus (EBV)-positive nasopharyngeal carcinoma		16	While DTH responses could be observed post vaccination in vivo, no LMP1-, LMP2- or adenovirus-specific T cell responses were detected in the peripheral blood. 3 from 16 patients had clinical responses.	PFS was 1.92 months (95% CI: 1.6–2.1 months) OS was 6.0 months (95% CI 2.73–11.7 months) 6-month survival rate: 50% 1-year survival rate: 19%	A phase II study evaluating the safety and efficacy of an adenovirus-ΔLMP1-LMP2 transduced dendritic cell vaccine in patients with advanced metastatic nasopharyngeal carcinoma	[215]
Autologous plastic adherent PBMC-derived dendritic cells transfected with a plasmid encoding both melan A and gp100 using the CL22 cationic peptide	N/A	N/A	metastatic melanoma	Phase I/II	25	expansion of effector responses to both antigens, to the human leukocyte antigen A2-restricted modified epitope, melan A ELAGIGILTV, and to a panel of MHC class I- and II-restricted epitopes	N/A	Gene Therapy Advisory Committee (study 084; 10 July 2003) and the South Birmingham Research Ethics Committee (203/298; 22 October 2003)	[216]
Autologous plastic adherent PBMC-derived dendritic cells; MART-1-reactive CD8+ TIL	MART-1 (26- 35 (27L); ELAGIGILTV	cyclophosphamide, fludarabine IL-2	metastatic melanoma	Phase II	18	well-tolerated	objective response rate was 30% (3/10) in the TIL arm and 50% (4/8) in the TIL + DC arm	NCT00338377	[217]
Autologous plastic adherent PBMC-derived dendritic cells-derived EVs	Mage3168–176. A1/B35; EVDPIGHLY; Mage3247–258. DP04,	N/A	stage III/IV melanoma	Phase I	15	There was no grade II toxicity. One patient exhibited a partial response. Exosome therapy promoted 2 stable diseases, 1 minor response, 1 partial response and 1 mixed response in skin or LN sites.	N/A	Vaccination of metastatic melanoma patients with autologous dendritic cell (DC) derived-exosomes: Results of the first	[218]

	KKLLTQHF VQENYLEY							phase I clinical trial	
Autologous plastic adherent PBMC-derived dendritic cells-fused to autologous tumor cells	tumor antigens from autologous tumor cells	IL-2	Stage IV melanoma	Phase I/II	25	There was OS advantage for NED stage IV patients, those receiving higher number of doses and increased frequency.	Median overall survival (OS) was 16.1 months with projected 5-year survival = 29%. Significant OS improvement for patients receiving ≥ 3 versus < 3 inoculations (43.1 vs. 16.7%, $p = 0.02$) was observed. Patients with no evidence of disease (NED) showed improved OS (80 vs. 14%)	NCT02301611	[219]
Autologous plastic adherent PBMC-derived WT1-encoding mRNA-electroporated dendritic cells; Immunomagneticly-isolated autologous CD14+ monocytes-derived WT1-encoding mRNA-electroporated dendritic cells	WT1	N/A	acute myeloid leukemia	Phase I	10	DC injections were well-tolerated; DC generation was tested	N/A	NCT00834002	[78]
autologous tumor-lysate-loaded DC, followed by TIL infusion	tumor antigens from 6 freezing/thawing cycles-lysed, 60 Gy-irradiated autologous tumor cells	cyclophosphamide	melanoma	Phase I	8	well-tolerated; one patient showed a complete remission	N/A	EU-nr 2008–000694–38	[220]

Autologous whole PBMC culture-derived dendritic cells	stimulated with TARP WT 27–35, TARP WT 29–37, and TARP EE 29–37 peptides	N/A	Prostate cancer	Phase I	41	TARP peptides administered as a Montanide/GM-CSF peptide emulsion or as an autologous peptide-pulsed dendritic cell; decreased Slope Log(PSA) compared to pre-vaccination baseline; Induced tetramer-positive T cells		NCT00972309	[114]
CD14-coated magnetic bead enriched monocyte-derived autologous dendritic cells	UV-b and gamma-irradiated adenocarcinoma cell line 1650	N/A	Non-small cell lung cancer	Phase I	16	Sno adverse effects observed; Tumor antigen-specific and independent immune responses	N/A	Dendritic Cell Vaccines for Non–Small-Cell Lung Cancer	[119]
Elutriation enriched autologous PMBCs were cultured in serum-free, non-adherent conditions in the presence of GM-CSF and IL-13	hTERT 988Y, HER2/neu 369V2V9, HER2/neu 689	cyclophosphamide	Advanced epithelial ovarian cancer (first or second remission)	Phase I/II	11	no grade 3/4 toxicities of 11, 2 patients relapsed during vaccination. among the 9 completing all 4 doses: 3 recurred at 6, 17, and 26 months; 6 remained disease-free at 36 months. Use of cyclophosphamide yielded a transient neutrophil drop and slight survival trend vs control (not statistically significant) Immune monitoring showed modest T cell responses to Her2/neu and hTERT by IFN-γ ELISPOT; diminished response to pneumococcal antigen suggests underlying immune suppression	3-year overall survival: 90%. Recurrences: 2 during vaccination; 3 within 6–26 months 67% remained disease-free at 36 months post-vaccination	Phase I/II randomized trial of dendritic cell vaccination with or without cyclophosphamide for consolidation therapy of advanced ovarian cancer in first or second remission	[221]
Elutriation enriched monocyte-derived autologous dendritic cells	PAP and PSA mRNA transfected	docetaxel	Castration-resistant metastatic prostate cancer	Phase II	43	No significant difference in PSA levels between docetaxel monotherapy and the combinatorial therapy	PFS and DSS were comparable: 5.5 versus 5.7 months (p = 0.62, log rank) and 21.9 versus 25.1 months (p = 0.60, log rank)	NCT01446731	[125]
Elutriation enriched monocyte-derived	Co-cultured with 10Gy	N/A	Metastatic melanoma	Phase II	42	Administration of DC was associated with longer survival at 5-year follow-	median OS was 43.4 versus 20.5 months (95%	NCT00948480	[222][223]

autologous dendritic cells	irradiated apoptotic autologous tumor cells					up evaluation Ex vivo matured DCs were more effective than direct vaccination with ATA and GM-CSF 70% reduction in the risk of death (hazard ratio = 0.304, *p* = 0.0053, 95% CI, 0.131 to 0.702)	CI, 18.6 to > 60 versus 9.3 to 32.3 months)		
Elutriation enriched monocyte-derived autologous dendritic cells	Co-cultured with 100Gy-irradiated autologous tumor cells	After radiotherapy and temozolomide	Glioblastoma multiforme	Phase II	11	No adverse events; Patients with the lowest combined immune activation response had shorter survival; 4 of the 5 patients in the immune response group, cluster 2, are alive after a follow-up of at least 2 years, whereas all 5 patients in cluster 1 have died; proportion of CD4+ IFNγ-producing cells, tumor-specific PF of CD8+ T cells, and proportion of CD8+ IFNγ-producing cells showed trends of an increase without statistical significance	median PFS was 9.5 months; median OS was 28 months	BB-IND 12903 and BB-IND 11162	[109]
Elutriation enriched monocyte-derived autologous dendritic cells	HER2	N/A	HER2/neu overexpressing ductal carcinoma in situ	Phase II/III	80	well-tolerated with only grade 1 and 2 toxicities observed; No evidence of remaining disease; median percent change in HER2/neu expression post-DC vaccine was minus 88% (i.e., a decline of 88%); 50%, (95% CI 28.2–71.8%) subjects achieved complete loss of detectable HER2/neu expression	N/A	NCT001070211	[116]
Elutriation enriched monocyte-derived autologous dendritic cells	HER2 via transduction	N/A	HER2-expressing metastatic	Phase I	33	objective response in 75% patients; 5–10 × 106 DCs 44% pts showed clinical benefits	N/A	NCT01730118	[117]

			solid cancer			Induction of anti-HER2 responses<br>Multifunctional responses over 70%			
Elutriation enriched monocyte-derived autologous dendritic cells transduced with the target antigens	granulocyte-macrophage (GM) colony-stimulating factor and carbonic-anhydrase IX (CAIX)	N/A	clear cell renal cell carcinoma	Phase I	15	Immune response measurements appeared more robust in higher dose cohorts, which appeared to be related to patients with stable disease at 3 months.	Of the 9 patients who received treatment, 1 expired of progressive disease, 2 patients were lost to follow-up and 6 patients are alive. Of the 6 patients, 5 have progressive disease, and 1 has completed treatment with stable disease at 27 months follow-up.	NCT01826877	[224]
Elutriation enriched monocyte-derived autologous type 1-polarized dendritic cells	HLA-A2–restricted peptides used in these studies were: ALPFGFILV (IL-13Rα2345–353:1A9V)23; TLADFDPRV (EphA2883–891)24; IMDQVPFSV (GP100209–217:M2)29; and SIMTYDFH	N/A	Malignant gliomas	Phase I/II	22	The regimen was well-tolerated;<br>no grade 3 or 4 toxicities;<br>The first four vaccines induced immune responses against GAAs<br>1 Complete response<br>41% progression-free for at least 12 months.	N/A	NCT00766753	[115]

	GA (YKL-40201–210). αDC1 were also loaded with the pan-DR epitope aKXVAAW TLKAAaZC								
Elutriation enriched PBMC-derived autologous dendritic cells	EF-1 (EWS/FLI-1) EF-2 (EWS/FLI-2) PXFK (PAX3/FKHR) HPV16E7	N/A	Metastatic or recurrent Ewing's sarcoma (ESFT) & alveolar rhabdomyosarcoma (AR)	Phase I	23	immunotherapy regimen was well-tolerated<br>Translocation breakpoint peptide–specific responses<br>E7-specific responses	5-year OS 31%	A Pilot Study of Consolidative Immunotherapy in Patients with High- Risk Pediatric Sarcomas	[139]
Endogenous DCs mobilised by FLT3 ligand treatment	N/A	N/A	Melanoma	Phase II	60	Increased peripheral monocytes and conventional DCs<br>Increases humoral and T cell responses<br>Activation of DCs, natural killer cells and T cells	N/A	NCT02129075	[151]
Ex vivo cultured DCs	autologous or allogeneic mesothelioma tumor cell line lysate		Mesothelioma	Phase I/II	29	promising long-term survival in the pooled cohort of the 3 studies	median OS was 27 months (95% CI: 21–47 months)<br>OS at 2 years was 55.2% (95% CI: 39.7–76.6%)<br>OS at 5 years was 20.7% (95% CI: 10.1–42.2%)	NCT02395679<br>NCT01241682<br>NCT00280982	[225]
Immunomagneticly-isolated autologous CD14+ monocytes-derived dendritic cells	tumor antigens from allogeneic	mitazalimab (agonistic	metastatic pancreatic cancer	Phase I	16	safe and well-tolerated;<br>systemic increase in activated and vaccine-specific T cell response;<br>increased T cell infiltration	N/A	NCT05650918	[82]

	mesothelioma tumor cell lysate from five unique, clinical-grade human multiple myeloma cell lines	CD40-specific antibody)							
Immunomagneticly-isolated autologous CD14+ monocytes-derived dendritic cells	tumor antigens from allogeneic mesothelioma tumor cell lysate from five unique, clinical-grade human multiple myeloma cell lines	N/A	Pancreatic ductal adenocarcinoma	Phase I	10	No vaccine-related serious adverse events; Seven out of ten patients have not experienced disease recurrence or progression at a median follow-up of 25 months All patients displayed a vaccine-induced response of Ki67+ and activated PD-1+ circulating T cells;	N/A	Netherlands Trial Register, NL7432	[226]
Immunomagneticly-isolated autologous CD14+ monocytes-derived dendritic cells	allogeneic tumor antigens from sonicated and 5 freezing/thawing cycles-lysed 2	Temozolomide and radiation therapy	newly diagnosed glioblastoma	Phase I	21	clinical benefit rate of 40%;	median PFS and OS of 9.7 and 19.0 months compare favorably to historical median PFS and OS with radiation and TMZ alone (6.9 and 14.6 months, respectively; progression-free survivor tail of 10% at 36 months	NCT01957956	[227]

	GBM cell lines						also compares favorably to historical PFS of 0.3% at 36 months with radiation and TMZ alone; prolonged survival in patients with MGMT promoter methylation both for median PFS (19.4 vs 9.4 months) and OS (26.5 vs 16 months); historical PFS and OS rates in MGMT promoter methylated (10.3 and 21.7 months) and unmethylated patients (5.3 and 12.7 months).		
Immunomagneticly-isolated autologous CD14+ monocytes-derived dendritic cells	tumor antigens from freezing/thawing-lysed, irradiated autologous tumor cells	neoadjuvant chemotherapy	colon cancer liver metastasis	Phase II	19	clear tendency to fewer and later relapses	median disease-free survival 25.26 months (95% CI 8.74-n.r versus observation arm median DFS 9.53 months (95% CI 5.32–18.88)	NCT01348256	[85]
Immunomagneticly-isolated bone marrow-harvested CD34+ cells-derived dendritic cells	tumor antigens from 5 freezing/thawing cycles-lysed autologous tumor cells	IL-2	metastatic renal cancer	Phase I/II	6	No adverse effect; transient and massive increase of circulating regulatory T cells (nTregs) possibly to IL-2;	N/A	Massive expansion of regulatory T cells following interleukin 2 treatment during a phase I-II dendritic cell-based immunotherapy of metastatic renal cancer	[228]

Immunomagneticly-isolated autologous CD14+ monocytes-derived chimeric, rimiducid-controlled CD40-transduced dendritic cells	human prostate-specific membrane antigen PA001	Rimiducid	advanced prostate cancer	Phase I	18	no dose-limiting toxicities. Immune upregulation as well as anti-tumor activity was observed with PSA declines, objective tumor regressions and robust efficacy of post-trial therapy	N/A	NCT01823978	[229]
Immunomagneticly-isolated autologous CD14+ monocytes-derived dendritic cells	HLA-A*2402-restricted, 9-mer WT1 peptide residues 235–243: CYTWNQMNL	N/A	advanced breast, ovarian, and gastric cancers	Phase I/II	10	clinical response was stable disease in seven patients; tumor shrinkage in three of seven patients;	N/A	ChiCTR-IPR-15005923	[230]
Immunomagneticly-isolated autologous CD14+ monocytes-derived dendritic cells	tumor antigens from allogeneic tumor lysate of five unique, clinical-grade human pleural mesothelioma cell lines	N/A	pleural mesothelioma	Phase II/III	176	MesoPher did not show improvement in overall survival in patients with pleural mesothelioma.	Median overall survival was 16.8 months in the MesoPher group and 18.3 months in the best supportive care group	NCT03610360	[231]
Immunomagneticly-isolated autologous CD14+ monocytes-derived dendritic cells	HLA-I and HLA-II personalized ligands	aspirin, gemcitabine/capecitabine,	non-metastatic resectable pancreatic adenocarcinoma	Phase Ib	3	The feasibility of identifying personalized neoantigens was demonstrated and an optimal manufacturing of incorporating long peptides into vaccine products was described.	N/A	CHUV-DO-0017_PC-PEPDC_2017	[232]

		nivolumab							
Immunomagneticly-isolated autologous CD14+ monocytes-derived dendritic cells	minor histocompatibility antigen (mHag)-peptide		multiple myeloma	Phase I/II	9	mHag-based donor monocyte-derived DC vaccination combined with DLI is safe, feasible and capable of inducing objective mHag-specific T cell responses	Five out of nine patients, of which four developed mHag-specific T cells, showed stable disease (SD) for 3.5–10 months.	ABR 39604; EudraCT: 2012–002435–28	[233]
Immunomagneticly-isolated autologous CD14+ monocytes-derived dendritic cells	HCV-specific CD8+ T cell epitopes	N/A	HCV-infected patients who failed conventional therapy	Phase I	6	Patients generated de novo responses, not only to peptides presented by the cellular vaccine but also to additional viral epitopes not represented in the lipopeptides, suggestive of epitope spreading. However, the responses were not sustained and failed to influence the viral load, the anti-HCV core antibody response and the level of circulating cytokines.	N/A	A phase I clinical trial of dendritic cell immunotherapy in HCV-infected individuals	[234]
Immunomagneticly-isolated autologous CD14+ monocytes-derived dendritic cells	idiotype protein or VDJ-derived class I-restricted peptides	N/A	Refractory multiple myeloma (patients previously treated with ≥ 2 lines)	Phase I/II	18	Idiotype-specific CD8+ T cell responses detected in 8/18 patients by IFN-γ ELISPOT and tetramer staining; increased frequency of activated CD8+ cells post-vaccination. No grade ≥ 3 toxicity; mild local reactions only. Clinical benefit limited but immune activation confirmed, subcutan injections of cryopreserved Id-pulsed DCs were safe and, in contrast with intravenous administrations, induced anti-MM T cell responses	Responses: 1 patient achieved partial response (paraprotein decreased with> 50%), 3 had minor response, 6 had stable disease (median duration 3–6 months), others progressed. Median OS: ~20 months from enrollment; median PFS: ~4 months	EudraCT 2009–016868–37	[235]
Immunomagneticly-isolated autologous CD1c+ conventional	gp100, tyrosinase, MAGE-C2,	N/A	resected stage		148	Functional antigen-specific T cell responses could be detected in 67.1% of patients tested	The 2-year RFS rate was 36.8% in the nDC treatment group and 46.9%	NCT02993315	[236]

and CD304+ plasmacytoid DCs	MAGE-A3 and NY-ESO-1		IIIB/C melanoma			in the nDC treatment group vs 3.8% of patients tested in the control group. No benefit in RFS was observed.	in the control group. Median RFS was 12.7 months vs 19.9 months, respectively.		
Undisclosed source of DCs transfected with tumor total mRNA or mRNAs that encoded hTERT and Survivin	N/A	N/A	high-risk prostate cancer	Phase I/II	20	broad antigen responses against several tumor antigens in response to personalized DC vaccines. This response was predominately associated with biochemical relapse free survival	11/20 were BCR-free over a median of 96 months (range: 84–99); The median time from the end of vaccinations to the last follow-up was 57 months (range: 45–60); All patients that developed BCR remained in stable disease within a median of 99 months (range: 74–99);	NCT01197625	[237]
Unspecified autologous monocyte-derived dendritic cells	MAGE-1, HER-2, AIM-2, TRP-2, gp100, and IL13Rα2	following radiotherapy	Glioblastoma multiforme	Phase II	124	OS benefit was statistically significant	median OS for IFNg responders was 433 days, while for a nonresponder it was 407 days	NCT01280552	[118]
Unspecified MNC-derived dendritic cell	recombinant human fusion protein composed of mucin 1 and glutathione S-transferase	N/A	Ovarian cancer	Phase II	56	Variable but measurable mucin 1-specific T cell response Progression free survival was not significantly longer	PFS between the CVac and SOC subjects (13 vs. 18 months) (HR = 1.18; CI 0.52–2.71; $p = 0.69$); PFS for second clinical remission patients treated with CVac ($n = 10$) was greater than 13 months; compared to the SOC control group ($n = 10$) was 5 months, an observed hazard ratio of 0.32 (CI 0.10–1.03; $p = 0.04$)	NCT01068509	[121]
